# Reversal of Stress‐Induced PIEZO1 Elevation with Mechanically Adapted Epicardial Patch for Myocardial Infarction Treatment

**DOI:** 10.1002/advs.202501663

**Published:** 2025-05-08

**Authors:** Yuwen Lu, Sibo Jiang, Ting Shen, Yuan Zhang, Chengbin He, Yun Gao, Liyin Shen, Qiao Jin, Yuting Zhao, Ping Liang, Chaochen Wang, Hongjie Hu, Jin He, Kaicheng Deng, Shuo Wang, Yunhe Chen, Jun Ling, Yang Zhu, Lenan Zhuang

**Affiliations:** ^1^ MOE Key Laboratory of Macromolecular Synthesis and Functionalization Department of Polymer Science and Engineering Zhejiang University Hangzhou 310027 China; ^2^ State Key Laboratory of Transvascular Implantation Devices Hangzhou 310009 China; ^3^ Institute of Genetics and Reproduction, Department of Veterinary Medicine College of Animal Sciences Zhejiang University Hangzhou 310058 China; ^4^ Key Laboratory of Cardiovascular Intervention and Regenerative Medicine of Zhejiang Province Department of Cardiology Sir Run Run Shaw Hospital Zhejiang University School of Medicine Hangzhou 310016 China; ^5^ Department of Radiology Sir Run Run Shaw Hospital Zhejiang University School of Medicine Hangzhou 310016 China; ^6^ Medical Imaging International Scientific and Technological Cooperation Base of Zhejiang Province Hangzhou 310016 China; ^7^ Key Laboratory of Combined Multiorgan Transplantation Ministry of Public Health the First Affiliated Hospital Zhejiang University School of Medicine Hangzhou 310003 China; ^8^ Institute of Translational Medicine Zhejiang University Hangzhou 310029 China; ^9^ Centre of Biomedical Systems and Informatics, ZJU‐UoE Institute Zhejiang University School of Medicine, International Campus Zhejiang University Haining 314400 China; ^10^ Digital Medical Research Center School of Basic Medical Sciences Fudan University Shanghai 200032 China

**Keywords:** biomechanical simulation, cardiac patch, myocardial infarction, PIEZO1

## Abstract

Elevated expression of the mechanosensitive ion channel PIEZO1 in response to abnormal mechanical stimuli is implicated in many diseases, including myocardial infarction (MI). However, no effective strategy is currently available to normalize PIEZO1 expression for disease management. This study investigates the therapeutic potential of mechanically adapted cardiac patches in reversing PIEZO1 elevation and treating MI. Increased mechanical stress and PIEZO1 upregulation are observed in ischemic cardiomyopathy myocardium. Using finite element analysis, elastomeric patches are designed and applied on MI rats to reduce left ventricular (LV) wall stress and mitigate LV remodeling. Molecular analysis reveals that patch treatment suppresses stress‐induced chromatin opening of the *Piezo1* promoter, reversing PIEZO1 elevation and restoring heart contraction gene expression. The patch's therapeutic benefits correlate with the reversal of PIEZO1 elevation is further validated in a porcine model. Notably, constant high expression of endogenous PIEZO1 partially blocks the patch's therapeutic effects, confirming that the mechanism of patch treatment involves reversing PIEZO1 expression, in addition to providing physical support. In conclusion, cardiac patches reduce LV wall stress, preserving cardiac function and geometry by both physically supporting and biologically reversing PIEZO1 expression, highlighting the potential of medical devices in normalizing PIEZO1 expression and treating related diseases.

## Graphical abstract

This work identifies fine‐tuning the expression of PIEZO1 as a critical molecular mechanism underlying the treatment of myocardial infarction by mechanically adapted cardiac patches, which can support the clinical translation of cardiac patch devices.

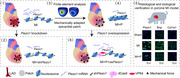

## 1 Introduction

Mechanosensitive cation channels are crucial for transducing physical stimuli from the cellular environment into biochemical responses in the human body.^[^ ^1^, ^2^ ^]^ Piezo1, a member of the Piezo family discovered in 2010,^[^ ^3^ ^]^ is expressed across various tissues and organs and plays a pivotal role in mechanotransduction across the cardiovascular,^[^ ^4^, ^5^ ^]^ hematologic,^[^ ^6^ ^]^ immune,^[^ ^7^ ^]^ and urinary^[^ ^8^ ^]^ systems. Pathologically elevated PIEZO1 expression has been linked to myocardial infarction (MI) in animal models,^[^ ^9^ ^]^ leading us to hypothesize that PIEZO1 levels would also increase in cardiomyopathy patients. Currently, there are no clinical treatments that effectively reduce PIEZO1 expression levels. Small molecule inhibitors, such as Dooku1^[^ ^10^ ^]^ and GsMTx4,^[^ ^11^ ^]^ which are being studied for this purpose, have only been validated in cellular models and small animal studies. Moreover, indiscriminate reduction of PIEZO1 expression could disrupt normal physiological functions in tissues and organs beyond the lesion area.^[^ ^12^ ^]^ Therefore, it is imperative to develop targeted strategies that selectively and precisely attenuate the high expression of PIEZO1 at the site of the lesion.

As a mechanoreceptor, PIEZO1 is responsive to intrinsic mechanical forces and activates downstream signaling pathways in a stress‐dependent manner.^[^ ^12^ ^]^ In severe diseases, such as heart failure^[^ ^13^ ^]^ and cancer metastasis,^[^ ^14^ ^]^ elevated PIEZO1 levels are attributable to increased mechanical stress within tissues, which is a consequence of structural pathologies. From the perspective of mechanical support provided by medical devices, orthopedic external bone fixators,^[^ ^15^ ^]^ and abdominal aortic stent grafts^[^ ^16^ ^]^ achieve therapeutic effects by reducing stress at the affected site and preventing structural damage to tissues directly in clinical application. Although it is not clear whether these devices function by reducing PIEZO1 expression, they demonstrate the significant benefits of mitigating tissue stress via medical devices.

Hence, we hypothesize that in MI with elevated PIEZO1 expression, the use of medical devices to reduce tissue stress and mechanical load can selectively suppress myocardial Piezo1 expression levels, thereby preserving left ventricular (LV) geometry and cardiac function. Following MI, loss of cardiomyocytes due to lack of oxygen supply leaves a necrotic, passively stretched region in the ischemic left ventricle.^[^ ^17^ ^]^ To compensate for the lost function of the necrotic myocardium, the border zone and remote myocardium exert greater contraction force, imposing higher wall stress in the injured myocardium,^[^ ^18^ ^]^ which promotes the formation of ventricular aneurysms^[^ ^19^ ^]^ or compensatory hypertrophic cardiomyopathy (HCM).^[^ ^20^ ^]^ Recent studies indicate that elevated PIEZO1 levels in the myocardium play a significant role in these processes in small animals and patients.^[^ ^4^, ^13^, ^20^ ^]^ In this study, combining the analysis of data from human, rats, and pigs, we found precise reduction of myocardial wall stress to normal levels with finite element simulation‐guided epicardial patch reversed the stress‐induced chromatin openness and high expression of *Piezo1* gene to the healthy levels, thereby preserving cardiac function and geometry. By knockdown and overexpression of Piezo1 in MI rats, the primary biological mechanism of the therapeutic effect of cardiac patches was identified as reverting stress‐induced Piezo1 expression, as a result of lowered cardiac wall stress. Together, we identified fine‐tuning the expression of PIEZO1 as a critical molecular mechanism underlying the treatment of MI by mechanically adapted epicardial patch, which has promising potential for the translational application of medical devices.

## 2 Results

### 2.1 Mechanical Stress and Expression of *PIEZO1* were Significantly Increased in the Myocardium of ICM Patients

**Figure 1:**
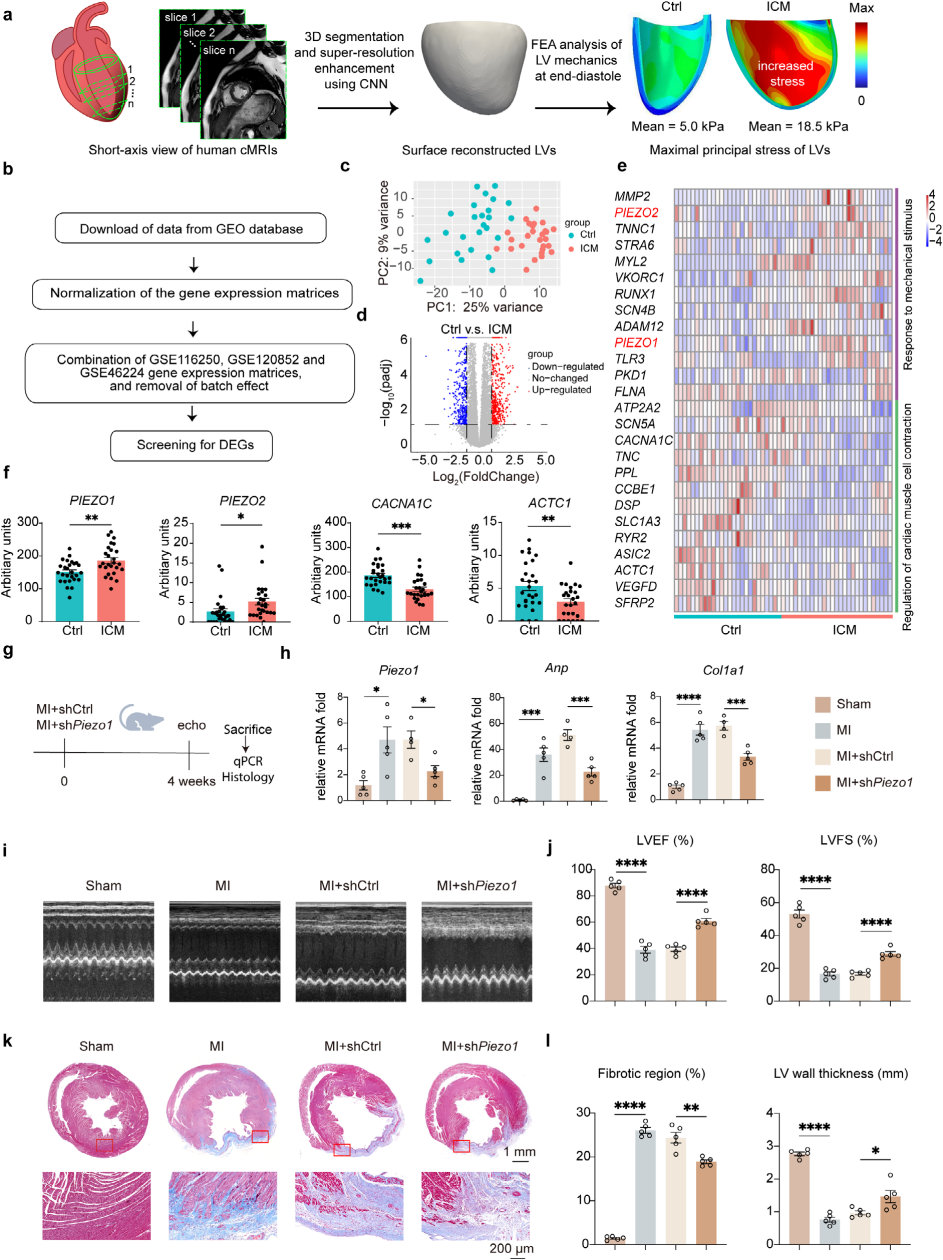
*PIEZO1* expression increased in the mechanical stressed myocardium of ICM patients and knockdown of it prevented ischemic injury in rats. a) Comparison of maximal principal stress distribution in the LV between healthy (Ctrl) and ischemic cardiomyopathy (ICM) hearts of human. b) Study design workflow of meta analysis. c) Principal component analysis (PCA) of merged RNA‐seq data showing the separation between control (Ctrl) and ICM individuals. d) Volcano plot of all expressed genes from merged RNA‐seq data. Horizontal dashed–dotted line represents the threshold for adjusted *p* value = 0.05, while vertical dashed–dotted lines show the Log_2_ (FoldChange) = 1. e) Heatmap presentation of the mechanical stimulus and cardiac muscle contraction related genes. f) Expression levels of *PIEZO1, PIEZO2, CACNA1C*, and *ACTC1* in the groups of Ctrl and ICM patients. g) Experimental design for *Piezo1* knockdown in the rat MI model. h) Expression levels of *Piezo1, Anp*, and *Col1a1* in the groups of Sham, MI, MI+shCtrl, and MI+sh*Piezo1*. i) Representative M‐mode echocardiography images from 4 groups, 4‐week postsurgery. j) Echocardiography analysis of left ventricular ejection fraction (LVEF), left ventricular fractional shortening (LVFS) in the four groups, 4 weeks after surgery. k) Masson's trichrome staining of heart sections from the four groups, 4 weeks after surgery (upper row). Scale bar = 1 mm. Representative local microscopic images of the border zone of LV (red boxes, lower row). Scale bar = 200 µm. i) Quantitative analysis of fibrotic region ratio and LV wall thickness. All data are presented as means ± SEM. *p < 0.05, **p < 0.01, and ***p < 0.001 comparison between groups was indicated in figures.

To measure the passive mechanical stress on the myocardium in human, the MRI data of healthy individual (Ctrl) and ischemic cardiomyopathy (ICM) patient were analyzed following the procedure in **Figure** **1**a. Specifically, cMRI slices of short‐axis were segmented and reconstructed into 3D models using Convolutional Neural Network (CNN), and finite element analysis (FEA) was performed to assess LV mechanics at end‐diastole. The results showed that in the Ctrl subject, the LV wall exhibited low maximal principal stress, with a mean value of 5.0 kPa. In contrast, the ICM patient demonstrated increased stress on the LV wall, with a mean maximal principal stress of 18.5 kPa, indicating severe mechanical stress in the infarcted myocardial tissue (Figure 1a), which was consistent with previous studies.^[^ ^21^ ^]^ To test whether the increased mechanical stress could induce the mechanosensitive gene expression in the myocardium of ICM patients, we combined and reanalyzed 3 ICM transcriptomic datasets (GSE116250, GSE120852, and GSE46224) from gene expression omnibus (GEO) database with batch effects removed (Figure 1b). The principal component analysis (PCA) showed that ICM significantly changed the transcriptome compared with the healthy control (Ctrl) (Figure 1c). Meanwhile, volcano plot visualized the differentially expressed genes (DEGs) including 1035 upregulated genes and 850 down‐regulated genes by comparing the ICM group with the Ctrl group (Figure 1d). Consistent with clinical observations, the expression levels of genes involved in the regulation of cardiac muscle cell contraction pathway represented by *CACNA1C* and *ACTC1* decreased in ICM patients (Figure 1e,f). Interestingly, the expression levels of genes related to mechanical stimulus represented by *PIEZO1* and *PIEZO2* significantly increased in the myocardial tissues of patients with ICM (Figure 1e,f). These results suggested that the expression levels of the genes related to mechanical stimulus including *PIEZO1/2* increased in the myocardium of ICM patients, which corelated with the higher wall stress in the injured myocardium.

### 2.2 Knockdown of *Piezo1* Expression in LV Myocardium Protected the Heart from Ischemic Injury in Rats

From the data of ICM patients, the expression of *PIEZO1* in heart was much higher than that of *PIEZO2*. We hypothesized that tuning down the mechanical stress stimulated expression of PIEZO1 could protect the myocardium from the ischemic injury. Three shRNAs targeting *Piezo1* were designed, sh*Piezo1*‐#1 which achieved 64% reduction in *Piezo1* mRNA levels in rat cells (Figure S1a, Supporting Information). Myocardial infarction (MI) surgery was performed on rats to generate an ICM model to test our hypothesis. MI group of rats experienced LV infarction induced by left anterior descending (LAD) ligation. Sham group, undergoing thoracotomy without ligation, served as control. Lentivirus expressing shRNA targeting *Piezo1* (sh*Piezo1*‐#1) was injected into the infarct myocardium of rats immediately post‐MI (MI+sh*Piezo1*). Lentivirus expressing scramble shRNA was injected to the same region as control (MI+shCtrl). Consistent with the observation in ICM patients, *Piezo1* expression significantly increased in the infarct myocardium of MI rats. The MI+sh*Piezo1* showed reversed *Piezo1* expression level to the similar level of Sham 4 weeks post‐MI (Figure 1g). The expression of *Anp* and some other inflammatory factors like *Il6r* and *Nlrp3* were downregulated in MI+sh*Piezo1* compared with MI+sh*Ctrl* (Figure S1c, Supporting Information). The myocardial fibrosis marker gene *Col1a1*, *Col3a1*, and *Tgfb1* were downregulated, correlated with the reversal of Piezo1 (Figure 1h; and Figure S1d, Supporting Information). Echocardiography images were used to measure the effects of reverting *Piezo1* expression on cardiac functions 4 weeks post‐MI (Figure 1i). In the MI+shCtrl group, LVEF and LVFS of rats were 39.4% and 16.7%, respectively. The reversal of *Piezo1* expression by knockdown improved the LVEF and LVFS to 60.8% and 28.8% (Figure 1j). Cardiac contractile function is related to calcium ions, and studies have shown that PIEZO1 could affect the changes in genes related to calcium ions. Knockdown of *Piezo1* caused a downregulation of *Camk2d* and upregulation of *Ryr2* (Figure S1e, Supporting Information). Masson's trichrome staining revealed similar results (Figure 1k,l), that the reversal of *Piezo1* expression reduced the infarction size to 18.9% and increased the LV wall thickness to 1.47 mm 4 weeks post‐MI, compared to 24.4% and 1.0 mm in the MI+shCtrl group. These data suggested that the stress‐induced increase of *Piezo1* expression in the infarcted myocardium involved in the cardiac remodeling post infarction and the reversal of stress‐induced *Piezo1* expression may be serve as a therapeutic target for protecting the ischemic heart.

### 2.3 Mechanically Adapted Epicardial Patch Provided Mechanical Support for the Ischemic Myocardium of ICM Patients and MI Rats

**Figure 2:**
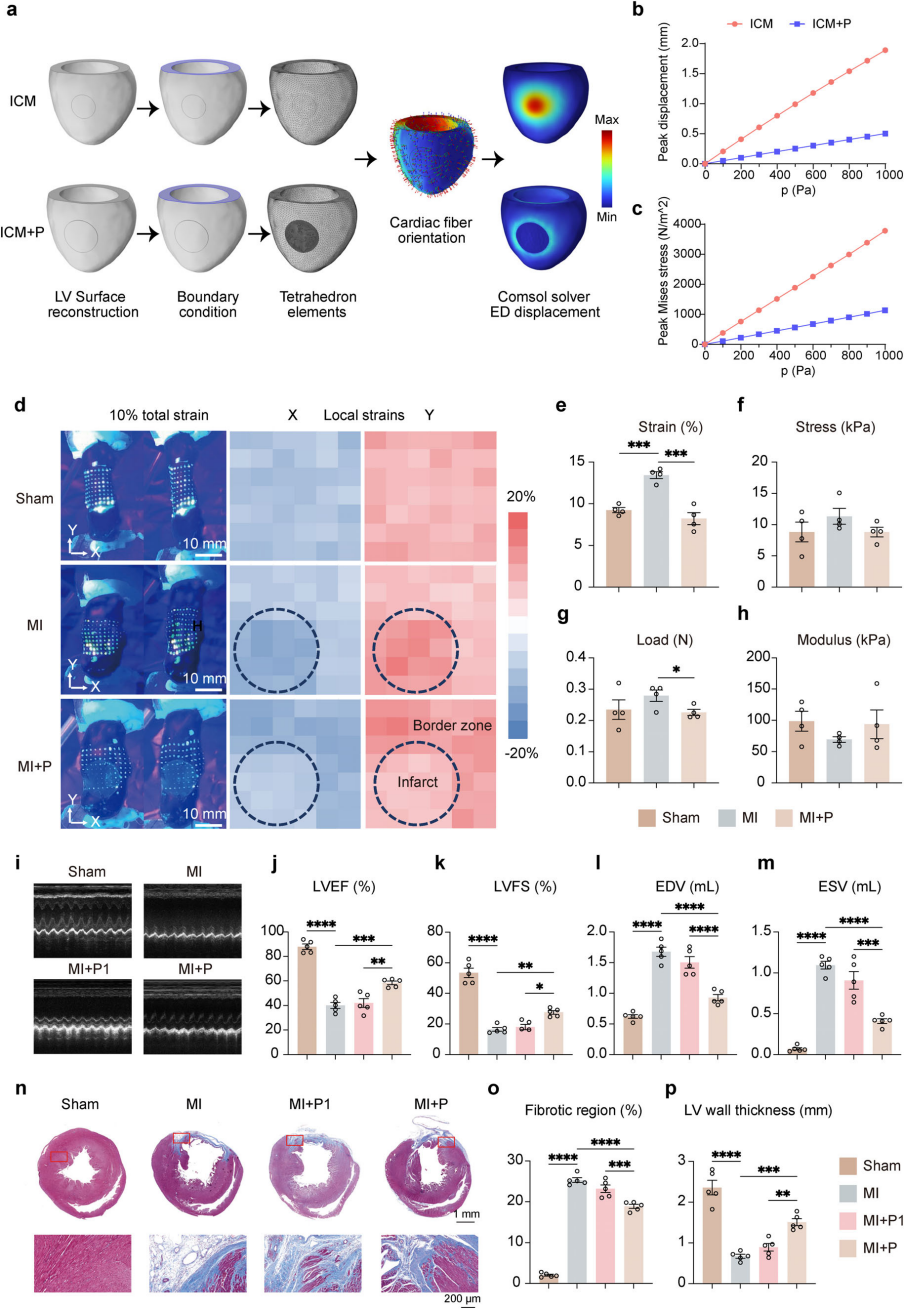
Mechanically adapted epicardial patch provided mechanical support for the ischemic myocardium of ICM patients and MI rats. a) Personalized biomechanical analysis of end‐diastolic LV mechanics from ICM patients. Simulated equibiaxial extension tests were conducted with a finite‐element model. b) Peak displacement‐pressure plots and c) peak Mises stress‐pressure plots of the infarct zone of LV in ICM patients and those treated with patches in the transmural direction. d) Images of excised myocardium before and after 10% uniaxial stretch along the circumferential direction (left column). The right column shows the local strain distribution in X (longitudinal, blue) and Y (circumferential, red) directions on stretched myocardium (Sham, MI, and MI+P) at 10% total strain. Scale bars = 10 mm. e) Strain, f) Stress, g) Load, and h) Composite modulus of the infarct region. i–p) Echocardiography analysis of j) left ventricular ejection fraction (LVEF), k) left ventricular fractional shortening (LVFS), l) end‐diastolic volume (EDV), and m) end‐systolic volume (ESV) 4 weeks after patch implantation. n) Masson's trichrome staining of the rat whole hearts from Sham, MI, MI+P1, and MI+P (upper row). Scale bar = 1 mm. Representative local microscopic images in the border zone (red boxes, lower row) 4 weeks after surgery. Scale bar = 200 µm. Quantitative analysis of o) ratio of fibrotic region and p) wall thickness in left ventricle. All data are presented as means ± SEM. *p < 0.05, **p < 0.01, and ***p < 0.001 comparison between groups was indicated in figures.

Stress‐induced elevated expression of PIEZO1 was connected to the passive stretch of the ischemic myocardium. By the methods of FEA^[^ ^22^ ^]^ and cMRI assessment^[^ ^23^ ^]^ using the ICM patient data (Xijing Hospital), we found that following acute myocardial ischemia, pressure, and volume overload lead to increased wall stress and strain consistent with previous studies.^[^ ^24^ ^]^ We hypothesized that if an implanted material could repress the stress‐induced PIEZO1 expression while unloading the mechanical stress. FEA was employed to investigate the efficacy of externally applied materials in redistributing LV wall mechanical stress, which could mitigate the outward expansion in the infarcted area of ICM patients. Further, we integrated a convolutional neural network with short‐axis cine‐cMRI of an ICM patient, applied automatic segmentation of ventricular structures and super‐resolution techniques, resulting in improved cardiac motion correction and enhanced segmentation quality. In this way, the LV of ICM patients (ICM group) was reconstructed ex vivo. In the LV model of ICM, a patch (20 mm diameter, 0.3 mm thickness, 50 MPa Young's modulus) was attached to the infarct and border zone, fully adhering to the epicardium, resulting in the reconstructed model labeled as LV and patch (ICM+P group). The stiffness, thickness and diameter of the cardiac patch can be adjusted according to the actual needs, where the dimensions should cover both the infarct and border zones. Finite element simulations were performed to assess the modified distribution of end‐diastolic displacement and von Mises stress throughout the LV wall following ICM and ICM+P (**Figure** **2**a), which were proportional to strain and stress in the direction of epicardium. Qualitatively, the interaction of the patch with the epicardium differentially altered myocardial displacement, strain and stress during the end‐diastolic process throughout the LV. The simulation results demonstrated that increasing the young's modulus and thickness of the patch enhanced its mechanical support on infarct expansion, thereby reducing LV transmural strain in the affected myocardium. Notably, when the patch modulus was within the range of 5–100 MPa, myocardial strain was nearly restored to the level observed in healthy myocardium. In contrast, patches with lower stiffness (e.g., 0.05 MPa) and smaller thicknesses (e.g., 0.3 mm) provided insufficient mechanical support and exhibited limited effectiveness in constraining infarct expansion (Figure S2, Supporting Information). At a representative modulus of 50 MPa, during passive relaxation to ED (*p* = 1000 Pa), the peak transmural displacement in the infarct zone surrounding the patch was 0.5 mm, representing a 73.5% reduction compared to the ICM group (Figure 2b). At the same time, in the ICM+P group, patch implantation resulted in a 70.0% reduction in von Mises stress at the epicardium (Figure 2c). The results demonstrated that mechanically adapted epicardial patches significantly alleviate stress and reduce myocardial deformation in ICM patients, restoring mechanical properties to levels comparable with healthy myocardium and offering a promising approach to mitigate myocardial expansion in the infarct area.

As confirmed by the above finite element simulations and previous studies, the treatment with biomaterial cardiac patch on the infarcted myocardium has been proven as an effective strategy.^[^ ^25^ ^]^ To release the mechanical stress posed on the infarcted myocardium, new polymer with larger Young's modulus was synthesized to generate elastic and stable biomaterial cardiac patch for providing mechanic support and releasing the stress on the injured myocardium to a greater extent. In Figure S3a (Supporting Information), [PCL‐*b*‐p(THF‐*co*‐CL)]*_m_* was synthesized via Janus polymerization of *ε*‐caprolactone (CL) and tetrahydrofuran (THF) as we previously reported.^[^ ^26^ ^]^ Nuclear magnetic resonance (NMR) shows that the percentages of CL and THF units in the copolymer are 73% and 27%, and THF separated PCL into relatively short segments, hence the copolymer has a low susceptibility to hydrolysis (Figure S3b, Supporting Information). The copolymer has a T*_m_* of 57 °C (Figure S3c, Supporting Information), and *M*_n_ of 203.0 kDa (Figure S3d, Supporting Information), thus at body temperature it is in rubber state. As a result, [PCL‐*b*‐p(THF‐*co*‐CL)]*_m_* is stable in simulated physiological condition. After degradation for 90 days, the mass remaining of [PCL‐*b*‐p(THF‐*co*‐CL)]*_m_* patch was 95.8%, showing a minimal weight loss (Figure S3e, Supporting Information). Therefore, the mechanical strength and elasticity of the patches were well maintained. Cyclic stretch test at physiologically relevant 10% strain showed that the patches maintained 95.7% original modulus (75.6 times of infarcted myocardium) after 90 d incubation in 37 °C phosphate buffered saline (PBS) (Figure S3f,g, Supporting Information). The 1000 times cyclic stretch test demonstrated that the patches maintained consistent stress–strain behavior with minimal hysteresis, confirming their durability and ability to provide sustained mechanical support to the myocardium (Figure S3h, Supporting Information). Additionally, the surface of the patch was smooth and planar to achieve intimate contact with the myocardium (Figure S3i, Supporting Information). These results supported that [PCL‐*b*‐p(THF‐*co*‐CL)]*_m_* patches could provide sustained stable mechanical support to myocardium and lower the mechanical stress in LV wall.

To evaluate the mechanical support provided by the cardiac patch, isolated rat hearts were stretched ex vivo. The LV myocardium was marked with fluorescent microneedle array, enabling the analysis of mechanical parameters.^[^ ^25^ ^]^ In the real measurement, when stretched at 10% strain along the circumferential direction of the myocardium, the healthy tissue in the Sham group (only thoracotomy operation, no ligation) had 9.3% strain in the circumferential (Y) direction and −9.0% strain in the longitudinal (X) direction (Figure 2d,e). In MI group (after 30 min of LAD ligation), the infarct area had greater strains in the circumferential (Y) direction (12.9%) and in the longitudinal (X) direction (−12.5%) (Figure 2d,e) compared to the healthy region on the same sample and the Sham control. In MI+P group (MI treated with patch secured and covered the infarct and border zone of the myocardium with 4 stitches), the cardiac patches sutured to the infarct tissue reduced circumferential strain to 7.6% and longitudinal strain to −7.8% in the infarct area (Figure 2d,e), smaller compared to the Sham group. As calculated from strain, total load on the myocardium samples, the stress and mechanical load in the infarct were reduced, and the modulus was increased as a result of patch implantation. Compared to the same myocardium before suturing the patches, cardiac wall stress in circumferential direction was reduced from 11.3 to 8.8 kPa (Figure 2f), mechanical load in the myocardium was reduced from 0.28 to 0.22 N (Figure 2g), and the complex modulus was increased from 69.5 to 93.8 kPa (Figure 2h). In MI+P group, due to mechanical compatibility between the patch and myocardium, the patch significantly reduced the stress and strain in the infarcted area to levels comparable to myocardium in Sham group, demonstrating its adaptability to the mechanical dynamics of the heart. The reduction in local strains after patch implantation was smaller in the remote area, compared to the infarct and border zone tissue covered by the patch, showing that the mechanical support effects mainly concentrated in the patched area. The observed increasing strain in border zone under the experimental condition reflects stress redistribution caused by the patch supporting the infarct zone. However, under physiological pressure conditions, the patch effectively reduces overall myocardial strain by reducing strain in the infarct zone, preventing adverse remodeling and protecting the heart from further damage.

Further, the cardiac patch's protective effect on the physiological function of hearts was verified in MI rats. To further decipher the important role of the mechanical support provided by the patch, but not the materials of the patch, the MI+P1 group of rats was included in, which was the MI rats treated with patches attached with only 1 stitch, sliding on the epicardium without mechanical support. Echocardiography images were applied to observe the restoration of patches on cardiac functions 4 weeks post‐MI (Figure 2i). LVEF and LVFS of rats from MI group were 40.0% and 16.5%, respectively. Patch implantation with 4 stitches (MI+P) maintained the LVEF and LVFS at 58.4% and 27.6%, respectively, significantly higher compared to MI and MI+P1 groups with the LVEF and LVFS at 42.0% and 18.0% (Figure 2j,k). In addition, the patch alleviated LV dilation. Hearts treated by 4 stitches patches retained significantly smaller end‐diastolic volume (0.9 mL, EDV) and end‐systolic volume (0.4 mL, ESV) compared to MI (1.7, 1.1 mL) and MI+P1 group (1.5, 0.9 mL) (Figure 2l,m). The mechanically adapted epicardial patch designed by FEA preserved cardiac function of MI hearts, maintaining performance levels very close to those of myocardium in the Sham group. Severe fibrosis (25.3%) was observed in the MI group (Figure 2n), as shown by Masson's trichrome staining. After 4 weeks of patch treatment, fibrosis area was 23.2% (MI+P1) and 18.9% (MI+P), significantly smaller compared to MI group (Figure 2o), and cardiac LV wall was significantly thicker in MI+P group (1.5 mm) than that in MI group (0.7 mm) and MI+P1 group (0.9 mm) (Figure 2p). The patch attached with 1 stitch detached from the myocardium, which failed to reduce the pathological LV remodeling or improve cardiac function compared to MI group. Together, these results showed that the mechanical support provided by the sutured patch, but not the 1 stitched patch, preserved cardiac function.

### 2.4 Patch Reverted Pathologically Elevated *Piezo1* Expression by Repressing Stress‐Induced Chromatin Opening in the Myocardium of MI Rats

**Figure 3:**
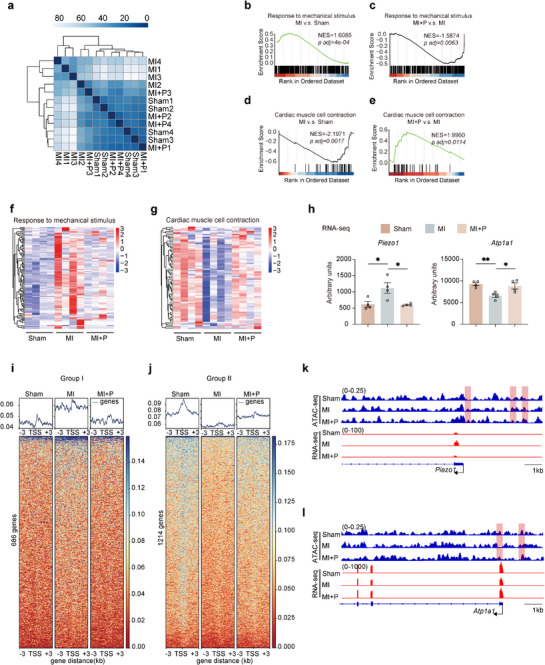
Patch reverted pathologically elevated *Piezo1* expression by repressing stress‐induced chromatin opening in the myocardium of MI rats. a) Correlation matrix analysis of transcriptomes from the infarct area in the Sham, MI, and MI+P (*n* = 4 per group). Darker colors indicate stronger correlations. b,c) Gene set enrichment analysis (GSEA) of the genes in response to mechanical stimulus between MI and Sham b) or between MI+P and MI c). d,e) GSEA of the genes in regulation of cardiac muscle cell contraction between MI and Sham d) or between MI+P and MI e). f) Heatmap for the expression of genes in response to mechanical stimulus. g) Heatmap for the expression of genes in regulation of cardiac muscle cell contraction. h) Arbitrary expression units for *Piezo1* and *Atp1a1* in the groups of Sham, MI, and MI+P in RNA‐seq assays. i,j) Patch effective peaks screened based on peak abundance in the promoter regions (−3 ≈3 kb) with Group I (Sum (MI)/Sum (Sham)>1.2 or Sum (MI)/Sum (Patch)>1.1) and Group II (Sum (MI)/Sum (Sham)<0.8 or Sum (MI)/Sum (Patch)<0.9). k,l) Snapshot illustrating ATAC‐seq and RNA‐seq peaks for *Piezo1* and *Atp1a1*, with or without patch treatment. All data are presented as means ± SEM. *p < 0.05, **p < 0.01, and ***p < 0.001 comparison between groups was indicated in figures.

To determine the therapeutic effect of the cardiac patch at the molecular level while the patch providing mechanical support to the infarct myocardium, RNA‐seq was applied to identify differentially expressed genes in the infarct regions of the three groups of rats (Sham, MI, and MI+P) 4 weeks post‐MI. Principal component analysis showed a separation in transcriptomes among the three groups (Figure S4a, Supporting Information). Consistently, the correlation matrix and clustering revealed the transcriptome features of MI+P and Sham groups were clustered together, separated with the MI group, which could reflect the patch treatment changed the transcriptome of the infarct myocardium to be similar with the Sham control (**Figure** **3**a). Compared to the Sham group, there were 2494 upregulated genes and 882 downregulated genes after MI (MI vs Sham) (Figure S4b, Supporting Information). Patch treatment resulted in upregulation of 392 genes and down‐regulation of 627 genes (MI+P vs MI) (Figure S4c, Supporting Information). In order to further explore the signal pathways changed after the treatment of patch, we analyzed a variety of gene sets by the gene set enrichment analysis (GSEA). Consistent with our finding in Figure 1, expression of genes in response to mechanical stimulus, including *Piezo1*, were significantly upregulated in MI compared with Sham (Figure 3b). Particularly, cardiac patch treatment reverted these elevated gene expressions in the infarct regions in the MI+P group (Figure 3c). Simultaneously, expression of genes in regulation of cardiac muscle contraction was significantly decreased in MI compared with Sham (Figure 3d), which consistent with the decreased heart contraction function in MI (Figure 3e). Nevertheless, patch treated infarct myocardium restored those cardiomyocyte contraction gene expression while preserving the heart function (Figure 3f,g). The RNA‐seq derived expression values of the representative genes, *Piezo1* for the response to mechanical stimulus; *Atp1a1* for the cardiac muscle cell contraction; were shown in Figure 3h. Similar results were confirmed by qRT‐PCR with total RNA extracted from the same area: *Anp* and *Piezo1* upregulated significantly after MI operation compared with Sham group, however the expression level of *Atp1a1* and *Camk2d* downregulated. Postimplantation patch, the abnormal expression of these genes was restored (Figure S4d, Supporting Information). *Nlrp3* and *Tnfa* for inflammatory response*, Col3a1, Tgfb1*, and *Timp1 for* collagen fibril organization were upregulation after MI and reversed to normal level via treatment by patch as well (Figure S4e–h, Supporting Information). Some ROS production and scavenging relative genes also significantly reflected by MI and patch treatment (Figure S4i, Supporting Information). These data suggested that the patch treatment could revert the stress‐induced expression of genes in response to mechanical stimulus including *Piezo1*, while providing mechanical support to the infarct myocardium. Moreover, the patch attached with only 1 stitch without mechanical support (MI+P1) could not revert the stress‐induced gene expression of *Piezo1* and *Anp* (Figure S4h, Supporting Information).

Recently, studies suggest that mechanical stress could transmit to the nuclear envelope, nuclear lamina, and chromatin from adhesion complexes via the linker of nucleoskeleton and cytoskeleton (LINC) complex; thereby, affect chromatin accessibility to regulate gene expression.^[^ ^27^, ^28^ ^]^ To gain further insights into the mechanism of how the expression of *Piezo1* was correlated with the mechanical stress, we analyzed the chromatin accessibility of the infarct myocardium from the three groups of rats (Sham, MI, and MI+P) 4 weeks post‐MI, using the assay for transposase‐accessible chromatin with high‐throughput sequencing (ATAC‐seq). Figure S5a (Supporting Information) showed the overall chromatin open regions in Sham, MI, and MI+P. The distribution of the open regions on chromosomes of each sample were shown as Figure S5b (Supporting Information). We used featureCounts to measure the open region numbers on all genes and compared with MI (MI/Sham and MI/Patch). A significant positive correlation was observed between MI/Sham and MI/Patch, indicating that Sham and MI+P have the similar effect on chromatin opening compared with MI (Figure S5c, Supporting Information). Among all the chromatin opening regions, two groups of them were further analyzed to look at the patch effect on the chromatin accessibility around the genes’ transcription start site (TSS). ATAC‐seq signals around the TSS of group I genes (686 genes) with stress‐induced and patch‐reverted chromatin opening were plotted in average profile and heatmap (Figure 3i). Some of the mechanical stimulus related genes, such as *Stat1*, *Map1b*, *Mir125b1*, and *Ccnb1*, which showed stress‐induced and patch‐reverted RNA expression (Figure S5d, Supporting Information), were included in the group I. The group II genes (1214 genes) had MI‐repressed and patch‐enhanced chromatin opening around their TSS regions (Figure 3j). Some of the heart contraction related genes, such as *Camk2d*, *Tnni3*, *Myh6*, and *Atp1a1*, which showed MI‐repressed and patch‐enhanced RNA expression (Figure S5e, Supporting Information), were included in the group II. Finally, we found that the patch treatment reverted the stress‐induced chromatin opening of *Piezo1* (Figure 3k), *Stat1* and *Map1b* (Figure S5f, Supporting Information) promoters which was highlighted in red. The chromatin accessibility of heart contraction genes, such as *Atp1a1*, *Tnni3*, and *Myh6*, were shown in Figure 3l; and Figure S5g (Supporting Information). These results suggested the mechanical stress posed on the infarct myocardium induced the chromatin opening around the promoter of *Piezo1*, thereby, activated the RNA expression of *Piezo1*. While, the patch treatment released the mechanical stress, decreased chromatin opening, and reverted the pathologically elevated expression of *Piezo1*.

### 2.5 Reverting Stress‐Induced *Piezo1* Expression by Patch Implantation Improved LV Function and Limited LV Remodeling in Porcine Model

**Figure 4:**
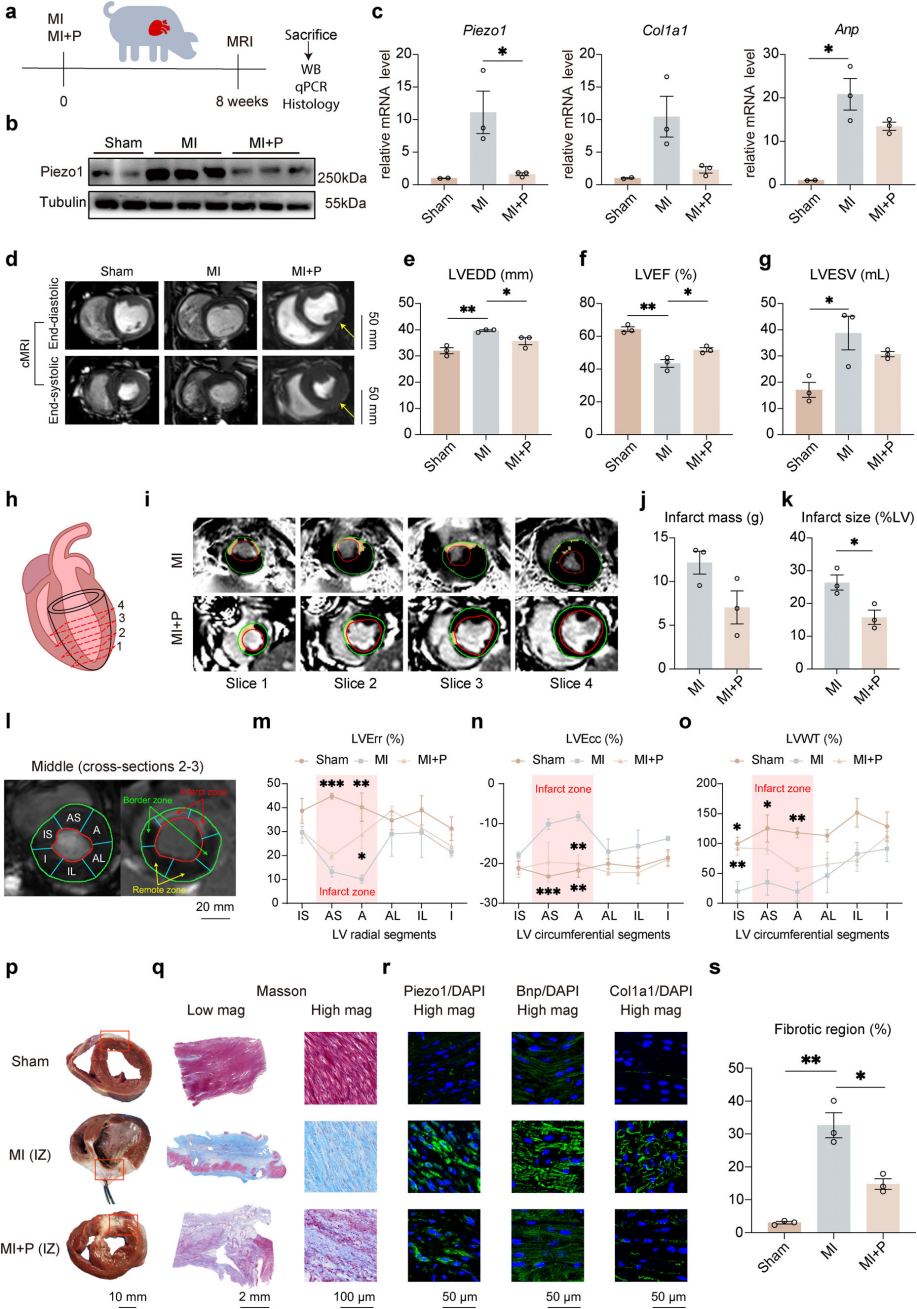
Reverting stress‐induced *Piezo1* expression by patch implantation improved LV function and limited LV remodeling in porcine model. a) Schematic of the pig experimental design including the groups of Sham, MI and MI+P. b) Immunoblot detection of Piezo1 from infarct zone of pig hearts of Sham, MI, and MI+P group. Tubulin was used as a loading control. c) Expression levels of *Piezo1, Anp, Col1a1* among Sham, MI, and MI+P group, analyzed via qRT‐PCR with RNA extracted from infarct zone of pig hearts 8 weeks post‐MI. d) Representative cMRIs at the end‐diastolic and end‐systolic phases at 8 weeks post‐MI. e–g) Quantitative analysis of LV end‐diastolic diameter (LVEDD) e), left ventricular ejection fraction (LVEF) f), and LV end‐systolic volume (LVESV) g) as assessed by cine‐cMRI (*n* =  3 biologically independent animals per group). h) Schematic representation of cMRI short‐axis slices, from apex to base (1–4). i) LGE‐cMRIs (from apex to base, 1–4) of two representative pig hearts analyzed at 8 weeks post‐MI (upper: MI; lower MI+P). The infarct area is counterstained in yellow. j,k) Infarct mass j) and size (calculated from LGE‐positive regions as a percentage of LV) k) at 8 weeks post‐MI. l) Schematic of LV segments. As shown in the illustration, the middle section (cross‐sections 2–3) was divided into six circumferential segments corresponding to the infarct zone, border zone, and remote zone. m–o) Measurements of LV Err m), LV Ecc n), and LVWT o) in the indicated groups (*n* =  3 biologically independent animals per group). p) Representative image of hearts from each group. q,r) Representative histological analysis of the infarcted myocardium among the treatment groups, featuring Masson's trichrome staining q) and immunofluorescence staining for Piezo1, Bnp, Col1a1 r). s) Quantitative analysis showing the percentage of the fibrotic region. All data are means ± SEM. *p < 0.05, **p < 0.01, and ***p < 0.001 comparison between groups was indicated in figures.

Systematic analysis and experiments in rat showed that cardiac patch could be a promising medical device for alleviating MI induced ischemic injury through reverting *Piezo1* expression. To further solidify this finding and test the feasibility of patch treatment, large animal experiments were performed in pigs. MI was induced in 6 pigs via occlusion of the LAD coronary artery. The pigs were randomly divided into two groups: one treated with a commercial polypropylene patch sutured into the LV infarct zone (MI+P), and the other left untreated (MI). To establish a baseline, a control group of sham‐operated animals was also included (Sham) (**Figure** **4**a). 8 weeks post‐MI, after functional analysis, the infarct, border and remote zone myocardium samples were collected. By qRT‐PCR and Western blot analysis of the infarct myocardium, we replicated the transcriptomic analysis results in rats, that *Piezo1* expression increased at the levels of both protein and mRNA 8 weeks post‐MI (Figure 4b,c). As a result of cardiomyocyte injury caused by MI, the heart failure marker gene *Anp* upregulated significantly, accompanied with the upregulation of myocardial fibrosis marker *Col1a1*. Interestingly, consistent with the results in rat, the patch treatment for 8 weeks in porcine MI model reverted the infarct *Piezo1* expression to the similar level in Sham (Figure 4c). At the same time, the expression levels of *Anp* and *Col1a1* downregulated to a lower level compared to MI, indicating the symptoms of ischemic injury were relieved (Figure 4c). Cardiac structural and functional assessments were performed using cMRI based on cine‐cMRI and LGE‐cMRI 8 weeks post‐MI. Representative cine‐cMRI images from three representative pigs per group revealed that LV adverse remodeling was significantly mitigated in the MI+P group compared to the MI group, as evidenced by the LV end‐diastolic diameter (LVEDD) (Figure 4d,e). LVEF also demonstrated a significant improvement following patch treatment compared to MI (Figure 4f). LV end‐systolic volume (LVESV) showed no significant differences between MI and MI+P, but still indicated a beneficial effect (Figure 4g). Moreover, representative LGE‐cMRIs of four short‐axis cross‐sections (1‐4, Figure 4h) of LV from two representative pigs per group at 8 weeks after MI are depicted in Figure 4i. The gadolinium‐retaining regions were counterstained in yellow, representing infarct mass. The infarct mass did not show a significant difference between MI and MI+P attributed to variations in individual weight (Figure 4j). However, the infarct size was observed to be nearly 50% smaller in pigs subjected to patch implantation compared with those in MI (Figure 4k). Additionally, cMRI enables the calculation of LV strain and wall thickness across different regions. The LV radial strain (LV Err) and circumferential strain (LV Ecc) were regionally evaluated in the middle sections (short‐axis cross‐sections 2–3, Figure 4l). In the infarct zone, regional analysis indicated significant LV Err and LV Ecc recovery in MI+P compared to MI 8 weeks post‐MI (Figure 4m,n). For the border and remote zone, despite the slight recovery of LV Err and LV Ecc in MI+P, no significant differences were observed when compared to MI, attributed to the small sample size. The regional analysis of LV end‐systolic wall thickening (LVWT) yielded similar results (Figure 4o). Moreover, it was demonstrated that in the border and remote zones, compared to the infarct zone, there was a substantial increase in the absolute values of these factors, particularly in MI. This suggests a compensatory thickening phenomenon in noninfarcted areas to maintain LV ejection fraction.

After cMRI examination, pigs were euthanized and hearts were collected for further analysis. Observation of LV short‐axis cross‐section myocardial tissue revealed the fibrotic area in the infarcted region was significantly reduced at 8 weeks post‐MI (Figure 4p). Masson's trichome staining of the LV sections from the infarct region demonstrated that at 8 weeks post‐MI, the infarcted regions in pigs treated with patch exhibited preserved distinct and thick muscle layers, akin to those observed in Sham (Figure 4q). Furthermore, immunofluorescence characterization of the myocardium infarct zone showed that the expression levels of Piezo1, Bnp, Col1a1 in MI+P were found to be similar to those in the Sham group, significantly lower than those in MI (Figure 4r; and Figure S6a, Supporting Information), which aligned with the differences observed in earlier qRT‐PCR analysis results among the three groups (Figure 4c). Additionally, the fibrous content in MI+P was half of that in the MI group, correlating with the expression level of Col1a1 (Figure 4r,s). Additionally, immunofluorescence results further demonstrated that in MI+P, the infarct zone and border zone exhibited improved cell organization and reduced fibrosis compared to the MI group, with Piezo1 expression significantly downregulated. The remote zone in MI+P also showed better structural integrity and lower Piezo1 expression (Figure S6b,c, Supporting Information). These observations in big animal experiments provided compelling evidence that patch treatment can effectively mitigate fibrosis and enhance cardiac remodeling post‐MI, which correlated with reverting the stress‐induced high expression of PIEZO1.

### 2.6 The Biological Mechanism Underlying the Therapeutic Effect of the Cardiac Patch was Primarily on the Reversal of *Piezo1* Expression

**Figure 5:**
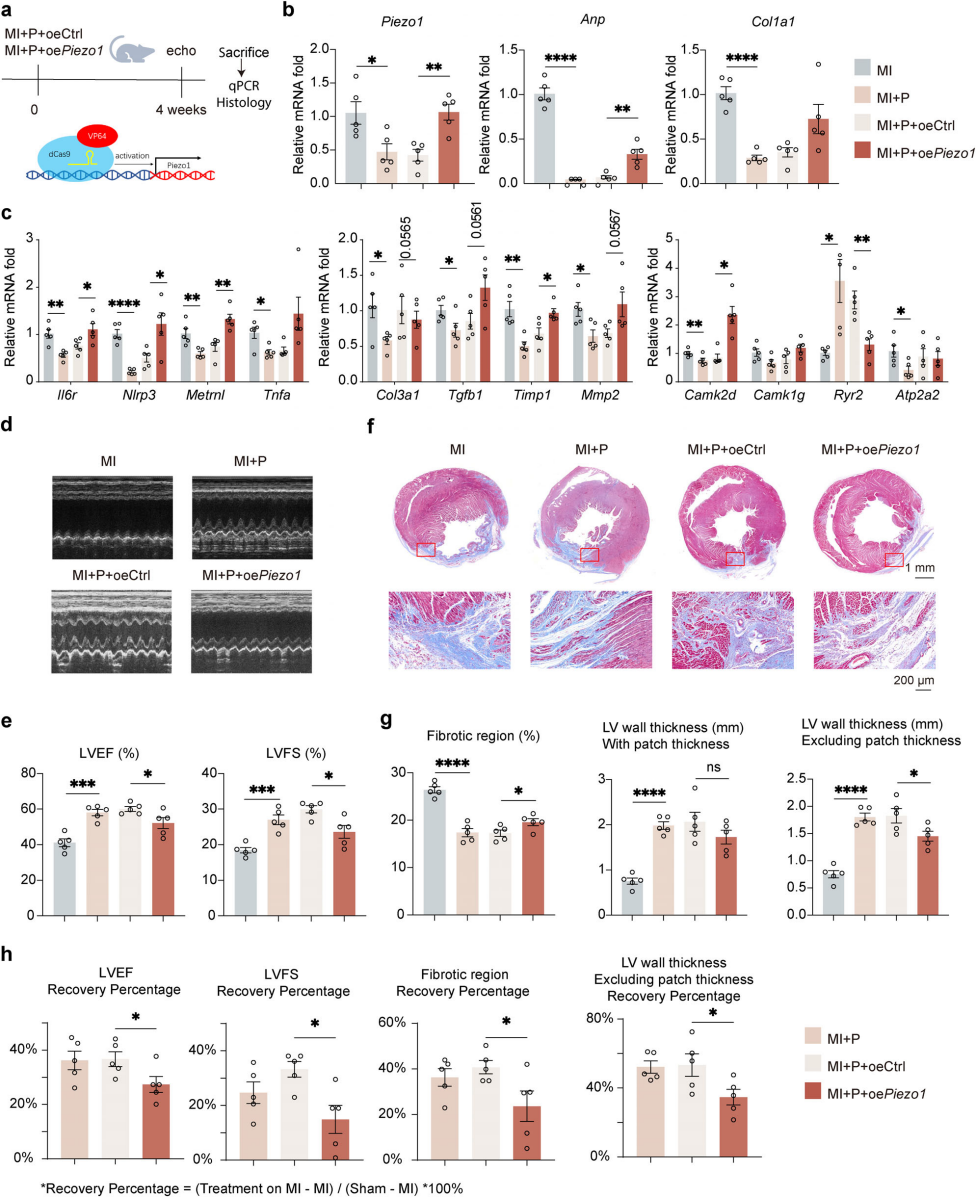
The biological mechanism underlying the therapeutic effect of the cardiac patch was primarily on the reversal of *Piezo1* expression. a) Experimental design of *Piezo1* overexpression in the rat MI model. b) Expression levels of *Piezo1*, *Anp*, and *Col1a1* in the groups of MI, MI+P, MI+P+oeCtrl, and MI+P+oe*Piezo1*. c) Expression levels of in inflammatory response genes (*IL6r*, *Nlrp3*, *Metrnl*, and *Tnfa*), collagen fibril organization genes (*Col3a1*, *Tgfb1*, *Timp1*, and *Mmp2*) and calcium ion transport genes (*Camk2d*, *Camk1* *g*, *Ryr2*, and *Atp2a2*) in the groups of, MI, MI+P, MI+P+oe*Ctrl*, and MI+P+oe*Piezo1*, determined by qRT‐PCR with RNA extracted from the rats. d) Representative M‐mode echocardiography images from 4 groups, 4‐week postsurgery. e) Echocardiography analysis of left ventricular ejection fraction (LVEF), left ventricular fractional shortening (LVFS) in the four groups, 4 weeks after surgery. f) Masson's trichrome staining of heart sections from the four groups, 4 weeks after surgery (upper row). Scale bars = 1 mm. Representative local microscopic images of the border zone of LV (red boxes, lower row). Scale = 200 µm. g) Quantitative analysis of fibrotic region ratio and LV wall thickness including or excluding patch. h) The recovery percentage of LVEF, LVFS, Fibrotic region, and LV wall thickness in MI+P, MI+P+oeCtrl, and MI+P+oe*Piezo1* group. Recovery Percentage = (Treatment on MI – MI)/(Sham – MI) *100%. All data are presented as means ± SEM. *p < 0.05, **p < 0.01, and ***p < 0.001 comparison between groups was indicated in figures.

The molecular mechanism underlying the therapeutic effect of the patch was correlated with reversal the stress‐induced *Piezo1* expression. We speculated that keeping a high‐level expression of *Piezo1* during the patch treatment could decrease the beneficial effect. Because of *Piezo1*’s cDNA is about 8221 bp, it is hard to overexpress the ectopic *Piezo1* using lentiviral system. CRISPR/dCas9‐based transcriptional activator system^[^ ^29^ ^]^ was chosen to activate endogenous *Piezo1* gene expression in the myocardium of MI+P rats (MI+P+oe*Piezo1*) (**Figure** **5**a). Three gRNAs targeting the promoter region of *Piezo1* were designed and their activation efficiency were tested. gRNA1 targeting the ATAC peak region on the promoter of *Piezo1* was selected for increasing the expression of *Piezo1* 60 times higher than control (Figure S1b, Supporting Information). Purified CRISPRa lentivirus targeting endogenous *Piezo1* were injected into the infarcted myocardium of MI rats treated with patch (MI+P+oe*Piezo1*), increased the endogenous *Piezo1* expression by about two folds than control (MI+P+oeCtrl) 4 weeks post‐MI, which was compatible with the increased expression level in MI (Figure 5b). We also check the variation of Piezo1 target gene. In the MI+P+oe*Piezo1* group, the upregulation of *Piezo*1 led to the reupregulation of inflammatory factor genes (like *Il6r, Nlrp3*, and *Metrnl*) and collagen organization genes including *Timp1* (Figure 5c) protected by the patch. Consistent with our speculation, the echocardiography 4 weeks post‐MI showed that the LVEF decreased to 52.1% in the MI+P+oe*Piezo1* group, compared with 58.0% in the MI+P group and 60.1% in the MI+P+oeCtrl group. Similarly, LVFS in MI+P+oe*Piezo1* was reduced to 23.6%, compared with 27.0% in MI+P and 30.0% in MI+P+oeCtrl (Figure 5d,e). Although the elevated *Piezo1* level did not significantly upregulate *Col1a1*, histological evaluations showed overexpression of PIEZO1 resulted in a larger infarction size (19.6%) and smaller LV wall thickness (1.7 mm) compared to MI+P (17.4%, 2.0 mm) and MI+P+oeCtrl (17.3%, 2.0 mm) (Figure 5f,g). The Recovery Percentage was defined to evaluate the cardiac functional and geometrical recovery efficiency (Sham = 100%; MI = 0%). Patch treatment partially restored cardiac contraction in LVEF (36.27%) and LVFS (24.69%), decreased the fibrotic region (36.22%), and increased LV wall thickness (52.17%). However, compared with MI+P+oeCtrl, overexpression of Piezo1 (MI+P+oe*Piezo1*) significantly reduced the recovery effects of the patch in LVEF (23.64%), LVFS (14.88%), fibrotic region (27.39%), and LV wall thickness (34.69%) (Figure 5h). These findings suggested that with the constantly high expression of endogenous *Piezo1* the cardiac patch treatment partially lost the therapeutic effect; however, the patch was constantly providing the physical supportive force to partially keep the contraction and geometry of the infarcted heart. Together, by interfering (Figure 1) and activating (Figure 5) endogenous PIEZO1, we proved that the biological mechanism underlying the therapeutic effect of the cardiac patch is mainly on fine tuning the expression of *Piezo1*, in addition to its physical mechanical support.

## 3 Discussion

Our study has established the critical role of PIEZO1 in the pathogenesis of ischemic cardiomyopathy (ICM), primarily driven by increased mechanical stress on the infarcted tissue. Previous studies have demonstrated elevated PIEZO1 levels in the animal models and patients with hypertrophic cardiomyopathy (HCM),^[^ ^20^ ^]^ ICM,^[^ ^4^ ^]^ and myocardial infarction (MI).^[^ ^30^ ^]^ Consistent with these findings, our analysis of transcriptomes from ICM patients revealed significantly increased expression levels of PIEZO1 in the myocardium (Figure 1). Furthermore, knockdown the stress‐induced high expression of PIEZO1 via shRNA in vivo protected the injured myocardium in rats (Figure 1). To further validate these findings, integrated approaches combining FEA, ex vivo measurements, and RNA‐seq analysis confirmed the correlations between reduced myocardial mechanical stress, normalized PIEZO1 expression levels, and therapeutic outcomes (Figure 3). These benefits were achieved through the implantation of patches that provided a stable and elastic supportive force (Figure 2). Additionally, our results revealed a relationship between mechanical stimulus and chromatin accessibility of the *Piezo1* gene loci (Figure 3), enhancing the understanding of the mechanisms by which mechanical stimulus affects PIEZO1 expression. Large‐animal experiments in pigs further confirmed the correlation of treatment outcomes from patches with the reversal of mechanical stress and PIEZO1 expression levels (Figure 4). Notably, CRISPRa‐mediated upregulation of PIEZO1 in the MI+P group negated the therapeutic effects of the patches, underlining the critical role of reverting Piezo1 in patch treatment (Figure 5). In summary, our study highlights the crucial role of PIEZO1 in cardiomyopathy therapy, showing that its targeted modulation can significantly improve treatment outcomes for MI.

Medical devices such as patches function by mechanically stabilizing specific regions of cardiac tissue, which indirectly modulates the activity of mechanosensitive PIEZO1 channels to restore them to normal levels.^[^ ^13^ ^]^ This ensures both safety and effectiveness of the treatment. The advantage of this approach is that it avoids the systemic effects typically associated with drug treatments^[^ ^10^, ^11^ ^]^ and the potential off‐target effects of gene therapy.^[^ ^31^ ^]^ Unlike small molecule drugs or gene editing, precise mechanical regulation of PIEZO1 expression through these devices minimizes the risks of excessively lowering PIEZO1 levels. PIEZO1 is crucial for maintaining various physiological functions.^[^ ^32^ ^]^ By using implantable medical devices, precise local stress reduction is achieved, which allows for accurate control over the spatial distribution of PIEZO1 levels. This location‐specific approach ensures that the physiological functions of PIEZO1 in other tissues and organs are not compromised. Furthermore, by adjusting the mechanical properties and degradation characteristics of the devices, in conjunction with FEA, the mechanical compatibility between the patch and the myocardium can be optimized, and personalized treatment designs can be developed.^[^ ^33^ ^]^ This allows for precise control over the intensity and duration of mechanical support. Similar mechanical support could be achieved from other devices, such as myocardial injection hydrogels.^[^ ^34^ ^]^ These devices can be implanted through minimally invasive surgery,^[^ ^25^, ^35^ ^]^ enhancing the safety of the treatment. In conclusion, the method of using medical devices like mechanical adapted cardiac patches enables targeted improvement of cardiac conditions by controllably restoring PIEZO1 levels through mechanical stimulus reduction without interfering with normal physiological functions, demonstrating significant potential and advantages in managing cardiac remodeling and mechanics‐related cardiac conditions. Pathologically elevated Piezo1 in infarct zone could lead to the activation of calcium influx, ROS production, inflammatory response, and collagen fibril organization, which eventually develops into heart failure.^[^ ^4^, ^5^, ^13^, ^17^ ^]^ We used virus to knockdown the elevated Piezo1 caused by MI could improve cardiac function via recovering the calcium influx, myocardial fibrosis, and inflammatory response. However, when we activated the expression of Piezo1 at the same time of patch treatment, MI‐induced the dysregulation of calcium influx, myocardial fibrosis, and inflammatory response was not improved to normal level. These observations strongly suggested the cardiac patch improved cardiac function via downregulating Piezo1 and its downstream pathways (calcium influx, ROS, myocardial fibrosis, inflammatory response) related gene expression.

The therapeutic strategy of using medical devices to modulate PIEZO1 levels presents promising prospects for clinical translation, suggesting a pathway for pioneering treatments in cardiac diseases. However, transitioning from experimental models to clinical practice entails several scientific and technical challenges. First, the biological mechanism by which mechanical stimulus variations influence the accessibility of the PIEZO1 coding region remains poorly understood. A more comprehensive understanding of this process is crucial, as it is directly related to the precise control of PIEZO1 expression. Without detailed mechanistic insights, the application of these devices might not achieve the desired specificity in modulating PIEZO1, leading to suboptimal clinical outcomes. Second, the timing of device implantation poses a significant challenge. It is known that Piezo1 expression is increased in infarct zone, border zone, and remote zone 4 weeks and 8 weeks post‐MI.^[^ ^5^, ^9^ ^]^ Typically, devices should be implanted after the cardiac condition stabilizes post‐MI.^[^ ^36^ ^]^ In this study, patches were applied following LAD ligation, at which time Piezo1 expression has not fully responded to the ischemic event,^[^ ^5^ ^]^ the patch treatment still reversed the stress‐induced Piezo1 expression in all myocardial regions including infarct zone, border zone and remote zone (Figure S6, Supporting Information). Third, the therapeutic efficacy of the patch is different between species. This difference could be attributed to the species‐specific physiological^[^ ^37^, ^38^ ^]^ and experimental conditions. The cardiac physiology, the size of the patch and the surgery procedures are different between rat and porcine models. Also, further investigation with optimized implantation timing in animal models is needed to enhance therapeutic outcomes. MI leads to an increase in mechanical stress, which in turn enhances the expression of Piezo1. Abnormally elevated Piezo1 then acts as a pathogenic factor, ultimately leading to heart failure.^[^ ^4^, ^5^, ^13^, ^17^ ^]^ Those two processes form a PIEZO1‐mediated positive feedback loop. We used the cardiac patch to protect cardiac function via alleviating mechanical stress and reversing the elevated Piezo1 as well as its downstream pathways.

Notably, ACORN trials showed that ventricular mechanical restraint alone failed to prevent heart failure progression or reduce mortality.^[^ ^39^ ^]^ The Acorn device (Acorn Cardiovascular, St. Paul, Minnesota), which circumferentially encloses both ventricles with a nondegradable mesh, imposes uniform constraint without regard to regional mechanics. In contrast, our patch design, guided by FEA, enabled tailored mechanical support for regional infarcted myocardium and yielded both functional and mechanistic benefits.

Furthermore, delayed implantation also appears effective: Wagner's group^[^ ^40^ ^]^ observed functional gains from PECUU patches implanted 8 weeks post‐MI, and our ongoing study shows improved systolic function with patches placed 2 weeks post‐MI, suggesting a viable therapeutic window despite persistent PIEZO1 upregulation. These findings raise important questions regarding the optimal timing for patch implantation. The therapeutic effect and alterations of Piezo1 expression of patch implantation at other time points post‐MI needs to be studied to specify the optimal time point for the patch implantation. In addition, a thorough investigation of the temporal dynamics of PIEZO1 in response to cardiac patches is required to further validate the efficacy of these interventions.

## 4 Experimental Section

### Data Availability

Detailed methods are provided in the Supporting Information Methods. All the sequencing data have been deposited in NCBI's Gene Expression Omnibus (GEO, http://www.ncbi.nlm.nih.gov/geo) and are accessible through GEO series accession number GSE202228. The data supporting the findings of this study are available from the corresponding author upon reasonable request.

### Myocardial Mechanics Simulation of the ICM Patient Before and After Patch Treatment by FEA

The cardiac magnetic resonance imaging (cMRI) data of ICM patients provided by Fudan University and Xijing Hospital (Xi'an, China) were employed to simulate the mechanics of the LV both before (ICM group) and after attaching a cardiac patch to the dilated epicardial region (ICM+P group) (approved by Fudan University Ethics Committee, FE22104R). The approach of LV 3D reconstruction aligns with previously established methods,^[^ ^21^ ^]^ as detailed in the Supporting Information Methods. The LV myocardium, excluding the infarct zone (the dilated epicardial region), was characterized as a hyperplastic material using the Holzapfel–Gasser–Ogden (HGO) model,^[^ ^41^ ^]^ which accurately depicted passive deformation during LV diastole. The myofiber angles at the base endocardium and epicardium of the LV were set at 60° and −60°, respectively, with 0° at the apex.^[^ ^42^ ^]^ These angles varied linearly across the heart wall and along the transmural direction. Both the infarct zone and the patch were modeled as isotropic materials. The infarct zone had a Young's modulus of 50 kPa,^[^ ^25^ ^]^ while the Young's modulus of patch was variably set within a range of 0.05–100 MPa, exceeding that of both healthy and infarcted myocardium, in order to evaluate the mechanical support provided by patches with different stiffness levels to the infarct region. The size of patch could be adjusted according to the MI area. To mimic the end‐diastolic relaxation, a pressure of 1 kPa is applied to the endocardium. Additionally, fixed boundary conditions applied to the basal surface of LV, ensuring the precision and reliability of the simulation results.

### Rat MI Model and Patch Treatment

All rat experiments were approved by the Guidelines of ZJU Animal Protection and Use Committee (20150119‐013). Male Sprague Dawley rats aged 8–10 weeks (200–240 g) were purchased from Zhejiang Academy of Medical Sciences, anesthetized with 1% pentobarbital by intraperitoneal injection. All rats were randomly divided into different groups. Rat hearts were exposed through thoracotomy. In the MI group, LV infarction was created by ligating the LAD coronary artery with a 6‐0 filament. In the MI+P group, immediately after infarction induction, a circular patch (8 mm in diameter) was implanted onto the infarcted myocardium below the ligation site. The patch was secured with four evenly spaced sutures along its perimeter to ensure stable fixation. In the MI+P1 group, the patches (8 mm diameter) were hung from epicardium with 1 stitch implantation after MI. Myocardium samples from Sham group (only thoracotomy operation, no ligation) was used as control group. Finally, the chest was closed with 3‐0 sutures. Rats were carefully monitored until they fully recovered from anesthesia. Rat cardiac function was assessed using echocardiography measurements, while myocardial mechanics were evaluated through stretch test ex vivo. Infarct zone tissue was used for histology and immunofluorescence analysis, and subsequent molecular experiments (RNA‐seq, ATAC‐seq, qRT‐PCR).

### Pig MI Model and Patch Treatment

All pig experiments were approved by the Institutional Animal Care and Use Committee of Zhejiang University (No. ZJU20210245). The pigs were transported from the production facility to the experimental facility and acclimatized for 2 weeks to the environment, with a 12‐h fasting period before surgery. Pigs were weighed and administered a combination of 6–8 mg kg^−1^ Zoletil 50 and 0.02 mg kg^−1^ atropine as a basal anesthesia by intramuscular injection. A 20G catheter was inserted into the auricular vein and secured to establish venous access, and the pigs were transported to the surgical table and placed in the prone position. A specially designed laryngoscope was used to expose the glottis and vocal cords, followed by the insertion of a 7.0–7.5Fr endotracheal tube. The pigs were then positioned supine and the anesthesia breathing circuit was connected, maintaining a respiratory rate of 16–20 bpm breaths per minute with a tidal volume of 10 mg kg^−1^. Muscle relaxation was achieved by intravenous injection of Pipecuronium Bromide at a dose of 0.1 mg kg^−1^, and anesthesia was maintained by inhalation of isoflurane. A sterile surgical field was established, and the skin of pig was disinfected. The sternum was incised along the midline, and subcutaneous tissue was sequentially dissected. A rib spreader was used to open the sternum, exposing the heart. The LAD coronary artery was identified, and permanent ligation was performed at the major branch approximately one‐third from the apex and after the first branch of the descending branch. In the MI group, the pericardium was sutured following ligation. In the MI+P group, 30 min after ligation, a circular patch (3 cm in diameter) was implanted onto the infarcted region below the ligation site. The patch was sutured with six evenly spaced stitches along its perimeter to ensure stable fixation, and then the pericardium was sutured. Myocardium samples from the Sham group, which underwent only a thoracotomy without ligation, served as the control. After thorough hemostasis, the chest was closed layer by layer. Penicillin powder was evenly distributed before each layer of suturing. A temporary drainage tube was inserted to completely aspirate air and blood from the thoracic cavity. After drainage, the tube was removed, and the incision was completely closed. The endotracheal tube was removed after completion of the surgery. Pigs were carefully monitored until they fully recovered from anesthesia. In the 8th week after surgery, cardiac function and myocardial mechanics of pigs from 3 groups were assessed via cMRI. Infarct zone, border zone, and remote zone tissues were used for histology and immunofluorescence analysis, and subsequent molecular experiments (qRT‐PCR, Western blot).

### Statistical Analysis

GraphPad Prism 8.0 was used for statistical analysis. Results are expressed as means ± SEM (standard error of the mean). Unpaired *t*‐tests were used to test the statistical differences from two groups. One‐way analysis of variance (ANOVA) was used for multiple comparisons. *p*‐value of <0.05 was considered statistically significant.

## Conflict of Interest

The authors declare no conflict of interest.

## Author Contributions

Y.L., S.J. and T.S. contributed equally to this work. Conceptualization: Y.Z., L.Z. Methodology: Y.L., S.J., T.S., C.H., L.S., J.H., S.W., Y.C., K.D. Investigation: Y.L., S.J., Y.G., Y.Z. Visualization: Y.L., S.J., T.S., Y.Z., Q.J., J.H. Supervision: Y.Z., H.H., J.L., L.Z. Writing—original draft: Y.Z., Y.L., S.J., T.S. Writing—review & editing: Y.Z., Y.L., S.J., P.L., C.W., L.Z.

## Supporting information



Supporting Information

## Data Availability

The data that support the findings of this study are available on request from the corresponding author. The data are not publicly available due to privacy or ethical restrictions.
