# Supplementary material for: Reversal of Stress‐Induced PIEZO1 Elevation with Mechanically Adapted Epicardial Patch for Myocardial Infarction Treatment
Source: Adv Sci (Weinh). 2025 May 8;12(27):2501663. doi: 10.1002/advs.202501663 (PMC12279209; doi:10.1002/advs.202501663)
Supplement: Supplementary file 1 — Supporting Information [file ADVS-12-2501663-s001.docx]

**Supporting information**

**Reversal of stress-induced PIEZO1 elevation with mechanically adapted epicardial patch for myocardial infarction treatment**

**Supplementary Methods**

**GEO data analysis**

The online human datasets, which comprised human left ventricular myocardium RNA-seq results from 27 non-failing (Ctrl) donors, and 26 (ICM) patients, were obtained from Gene Expression Omnibus (GEO, Accession No.: GSE116250, GSE120852 and GSE46224). GSE116250 includes samples from 14 non-failing donors and 13ischemic patients. GSE120852 includes samples from 5 control individuals and 5 end-stage ischemic patients. GSE46224 also includes samples from 8 non-failing donors and 8 ischemic patients. Detailed information about the databases were shown in **Supplementary Table 1.** The downloaded sra-formatted files were first converted to fastq format using fastq-dump command. Then, these files were processed using the same protocol to the rat's data described belowFastp was used to generate clean data by removing low-quality reads, poly-N reads, and adapter sequences. Then, the clean data of rat and human were aligned to their respective reference genomes (rat genome version 6.0.104, human genome version GRCh38) using Hisat2 tool ^[1]^. Subsequently, the samtools ^[2]^ was employed to transform the aligned data to sorted bam files, which were counted by featureCounts function from Rsubread package.

For humans, the raw counting matrices were processed and reclassified to have four groups: Ctrl, ICM for rats, batch effects were included in the normalization function. DESeq2 ^[3]^ was utilized to normalize these data, and the differentially expressed genes (DEGs) were defined as those genes which had adjusted *p* value < 0.05. The expression levels of *PIEZO1* and *PIEZO2* genes of human and rat extracted from the normalized data were analyzed by one-way ANOVA and Tukey's HSD post hoc. Then, principal component analysis (PCA) and heatmap were depicted after removing batch effect by removeBatchEffect of limma package ^[4]^ and stabilizing variances by vst function ^[3]^.

Gene ontology (GO) analyses of differentially expressed genes were carried out by the Database for Annotation, Visualization and Integrated Discovery (DAVID). To further explore the functional annotations of the DEGs, gene set enrichment analysis (GSEA) was conducted using the clusterProfiler package, and the gene sets related to mechanical stimulation and cardiac contraction were included in the analysis.

**Human cardiac MRI data collection**

We collected human cMRI data for cardiac mechanics simulation. The cMRI data of the heathy control individual (Ctrl, **Fig. 1a**) was obtained from the open-source dataset (https://data.mendeley.com/datasets/pw87p286yx/1) provided by Hammersmith Hospital (London, UK). The cMRI data of an ICM patient (ICM, **Fig. 1a**) was provided by Fudan University (Shanghai, China), while the cMRI data of another ICM patients (ICM, ICM+P, **Fig. 3a**) was provided by Xijing Hospital (ID: P010, Xi’an, China). All cMRI data underwent segmentation and reconstruction of LV for myocardial mechanics simulation.

**Segmentation and reconstruction for** **cardiac mechanics analysis from cMRI data**

Cardiac MRIs were evaluated by the independent Shanghai Key Laboratory of Medical Imaging Computing and Computer Assisted Intervention. The bi-ventricular structure of patients was reconstructed using bSSFP cine-cMRI, focusing on the early-diastole frame. A convolutional neural network (CNN) automatically created masks for the left and right ventricles in the short-axis MRI images. Radiologists then checked and refined these segmentations for accuracy. To further enhance the segmentation, a CNN-based super-resolution technique was used to achieve a detailed resolution of 2×2×2 mm, improving on the usual resolution of short-axis images (~2×2×10 mm). The LV epicardium was generated using Marching Cube algorithms and was sealed at the base to meet the requirements for finite element analysis.

**Myocardial mechanics simulation of** **a healthy** **individual and an ICM patient by FEA**

The cMRI data of a healthy individual provided by Hammersmith Hospital (London, UK) and the cMRI data of a ICM patient provided by Fudan University (Shanghai, China) were employed to simulate and compare the mechanical behavior of the LVs. The approach of LV 3D reconstruction and myocardial mechanics simulation align with previously established methodologies ^[5, 6]^.

**Synthesis of multiblock copolymers**

Tetrahydrofuran (THF, Sinopharm Chemical Reagent, China) was refluxed over potassium to remove the moisture. Propylene oxide (PO, 98%, Energy Chemical, China) and ε-caprolactone (CL, 99%, Acros, China) were distilled over CaH_2_ (Sinopharm Chemical Reagent, China) and stored in argon. Lu(OTf)_3_ was synthesized by hydrothermal reaction of Lu_2_O_3_ (>99.99%, Founde Star Science & Technology, China) and triflic acid (99%, Energy Chemical, China) as reported^[7]^ and dried in vacuum at 200 °C for at least 40 h.

[PCL-*b*-p(THF-*co*-CL)]_m_ was synthesized via Janus polymerization as previously described^[8]^. Lu(OTf)_3_ (0.3732 g, 0.6 mmol) was added into a flame-dried ampoule and dissolved in THF (8.35 g, 116 mmol) and CL (6.84 g, 60 mmol). PO (52.2 mg, 0.9 mmol) in THF (0.29 g, 4 mmol) was then added to initiate the polymerization. The ampoule was sealed and kept at 25 °C for 14 days. The crude product was precipitated in cold methanol, filtrated and dried under vacuum.

**Characterization of the copolymer**

Molecular weights and dispersities (Đ) were determined by size-exclusion chromatography (SEC) on Waters-150C equipment with Waters Styragel HR3 and HR4 columns and a Waters 2414 refractive index detector. THF was used as the eluent at a flow rate of 1.0 mL/min at 40 °C. Commercial polystyrene samples were used as calibration standard. Nuclear magnetic resonance (NMR) spectra were recorded on a Bruker Avance DMX 400 spectrometer (^1^H: 400 MHz). CDCl_3_ was solvent and tetramethylsilane (TMS) was internal reference. Analyses was performed by differential scanning calorimetry (DSC) on a Q200 TA Instrument. Samples were heated from -40 °C to 80 °C at a heating rate of 10 °C/min under nitrogen, held for 5 min to erase the thermal history, and then cooled to -40 °C and held for another 5 min. The second heating scan from -40 °C to 80 °C at the same heating rate was recorded.

**The morphology characterization of [PCL-*b*-p(THF-*co*-CL)]_m_ patch by SEM**

The patch sample was mounted on conductive tape and sputter-coated with gold for 30 seconds. Imaging was performed using an S-4800 SEM (Hitachi, Japan) at an accelerating voltage of 15 kV.

**Fabrication of the [PCL-*b*-p(THF-*co*-CL)]_m_ patches**

[PCL-*b*-p(THF-*co*-CL)]_m_ patches were dried from 15 wt% THF solutions in Teflon molds. THF was slowly evaporated at room temperature for 48 h. After the film was removed, 8 mm patches were cut using a hole punch.

**Safety Evaluation of the [PCL-*b*-p(THF-*co*-CL)]_m_ patches**

[PCL-*b*-p(THF-*co*-CL)]_m_ patches meet the characteristics of Class III medical devices. Safety assessments were conducted by the Chinese Academy of Inspection and Quarantine (CAIQ) following the standards outlined in GB/T 16886 and ISO 10993. The evaluations included the following tests: in vitro cytotoxicity, in vitro chromosome aberration, bacterial reverse mutation, acute oral toxicity in mice, skin sensitization, pyrogenicity, and hemolysis (in vitro method). No safety concerns were identified in any of these tests.

**Degradation rate of [PCL-*b*-p(THF-*co*-CL)]_m_ patches**

[PCL-*b*-p(THF-*co*-CL)]_m_ patches (8 mm diameter) were weighed (W_0_), then incubated in PBS at 37 °C, shook at 100 rpm (n = 3). Samples were rinsed 3 times with deionized water, freeze-dried for 1 day before recording the weight (W_1_) and change the PBS solution once every month. Mass remaining (%) was calculated as W_1_ / W_0_ × 100%.

**Mechanical property measurement of [PCL-*b*-p(THF-*co*-CL)]_m_ patches**

All cyclic stretch tests were conducted by stretching the samples to a strain of 10%, and releasing back to 0% strain for cycles by an Instron 5540A universal testing machine. Mechanical properties of the patch samples before and after 90-day degradation were measured under 10 times of cyclic stretch, at a stretching speed of 10 mm/min. Stress-strain behavior of patch samples under 1000 times of cyclic stretch was measured at a stretching speed of 50 mm/min.

**LV assessment with echocardiography measurement in rat MI model**

After 4 weeks of patch treatment in rat MI model, cardiac function was assessed using an ultrasound system (VEVO2100 ultrasound system, Visual Sonics, Canada). Left ventricular ejection fraction (LVEF), left ventricular fractional shortening (LVFS), end-diastolic volume (EDV) and end-systolic volume (ESV) were calculated. After echocardiography images was recorded, the LV myocardium was excised.

**Mechanical support by the patches on infarcted myocardium of rat ex vivo**

Rats were divided into Sham, MI, and MI+P groups as described in the Methods section. After 30 minutes of LAD ligation, （for the MI+P group, patch was sutured on the myocardium immediately after the LAD ligation）the LV myocardium was excised, circumferentially unfolded, and marked using a microneedle array seal with cone-shaped microneedles spaced 1 mm apart in both the X and Y directions. The microneedles were pre-dipped in fluorescent dye (Xiucai Chemicals, China) to transfer a dot array onto the epicardium. The microneedle array was 3D printed using photosensitive resin (BMF Precision Tech, China). LV myocardium samples from all groups were clamped onto an Instron universal mechanical testing machine and stretched to 10% strain at a rate of 1 mm/s. Patched myocardium samples underwent the same procedure. Stretching was performed under UV light, and the displacement of fluorescent dots was recorded with a fixed camera. Images captured before and after stretching were used to calculate local strains in the circumferential and longitudinal directions, and strain distribution was visualized using color-coded maps. The effects of patch treatment on stress, circumferential and longitudinal strain, mechanical load on the infarcted myocardium, and the modulus of the myocardium/patch composite at 10% stretching were calculated. Detailed measurement procedures are provided in our previous research ^[9]^.

**LV assessment with cMRI in pig MI model**

Cardiac magnetic resonance imaging was performed at 8 weeks after surgery. Before cMRI, it was requested to fast pigs for 12 hours and restrict water intake for no more than 6 hours. Additionally, it needed to administer intramuscular atropine 15 minutes before anesthesia to reduce airway secretion. Intramuscular injection of Zoletil R50 at a dose of 0.1 ml/kg is also requested. An indwelling needle should be left in a vein at the ear margin, and propofol should be injected intravenously at a rate of 3-5 ml per time. All ferromagnetic foreign bodies should be removed from pigs. They should be placed in a supine position with their head forward. An 18-channel phased-array abdominal coil should be used to cover the entire cardiac scanning area. An electrocardiographically gated technique should be employed, triggering image acquisition at the end-diastole of the heart.

Siemens 3.0T magnetic resonance (MAGNETOM Skyra, Siemens Healthineers AG, Erlangen, Germany) was used as CMR scanning equipment. The scanning protocol comprised localization phases (axial, coronal, and sagittal), cine sequences, T1-weighted sequences, T2-weighted sequences, resting first over-perfusion sequences, and late gadolinium enhancement (LGE) sequences. The cine sequence and LGE sequence acquired the standard short-axis position of the two-chamber heart, the four-chamber heart, and the whole left and right ventricles (layers 9-12). The T1-weighted and T2-weighted sequences were acquired in the standard short-axis position of the left ventricle only. The resting first-pass perfusion consisted of four layers, including the apical, midventricular, and basal short-axis positions, as well as the four-chamber heart. The cine sequence included the two-, three-, and four-chamber hearts, the LV outflow tract, and LV short-axis position. The cine images were acquired with the following parameters: layer thickness of 8 mm, layer spacing of 0 mm, repetition time (TR) of 3.51 ms, echo time (TE) of 1.54 ms, flip angle (FA) of 48°, an acquisition matrix of 256×204, and a field of view (FOV) of 340×320 mm2. LGE images were acquired 6-8 minutes after intravenous injection of 0.3 ml/kg of gadolinium diethylenetriamine pentaacetate (Gd-DTPA) (Beilu, Beijing, China) at an injection rate of 3 ml/s. The images covered the short axis of the right and left ventricles. The layer thickness was 8 mm, and the layer spacing was 0 mm. The repetition time (TR) was 9.24 ms. The cMRI scan was performed with the following parameters: a TE of 1.24 ms, an FA of 40°, an acquisition matrix of 256×184, and an FOV of 340×320 mm^2^. The acquisition matrix was 256×184 and the FOV was 340×320 mm^2^.

**Histological assessment**

For rats, the hearts were collected on 4 weeks. For pigs, the hearts were harvested 8 weeks post-surgery, with samples taken from the infarcted zone, border zone, and remote zone of LV (MI, MI+P). For Sham hearts of pigs, samples were collected from a corresponding site of LV (Sham). In all quantifications, we considered that the same regions were chosen in animals from different groups. The hearts were collected and stopped by potassium chloride solution. Then the hearts were fixed in 4% paraformaldehyde, embedded in paraffin and consecutively sectioned into several sections on the short axis at a 4-μm thickness. For histological analysis, LV geometry and fibrosis were assessed by Masson’s trichrome staining, with red areas representing muscle and blue areas representing collagen. Histological images were obtained using a digital slicing scanner (Olympus, VS200, USA). After Masson’s trichrome staining, fibrotic area (%) was calculated by the following formula: (total fibrotic area/total LV circumference area) × 100%, and the wall thickness of the scarred tissue was measured. Data were analyzed using ImageJ software.

**Immunofluorescence staining**

The pig hearts were harvested 8 weeks post-surgery, with samples taken from the infarcted zone, border zone, and remote zone of LV (MI, MI+P). For Sham hearts of pigs, samples were collected from a corresponding site of LV (Sham). For immunofluorescence staining, pig heart sections were fixed in 4% formaldehyde for 15 minutes, permeabilized by 0.2% Triton X-100 for 15 minutes, and then blocked in 5% BSA in PBS for one hour. The immunofluorescence was performed using anti-Piezo1 (1:300, Protein Tech, 15939-1-AP), anti-Col1a1 (1:500, CST, 72026S), anti-Bnp (1:500, Abclonal, a2179) as primary antibody overnight. And the slides were next stained with secondary antibody (1:1000, Yeasen, 33106ES60). After washing used cold PBS, stained sections were mounted with DAPI. Amplified fluorescence images were acquired using a LSM 880 confocal microscope (Carl Zeiss, Germany).

**RNA-seq experiment**

We extracted total RNA of tissues of the infarct zone from MI and MI+P groups in rats 4 weeks post patch treatment using TRIzol (Invitrogen, USA). In the Sham group, tissues from the similar LV site in the MI group were used. A total amount of 1 μg RNA per sample was used as input material for the RNA sample preparations. Sequencing libraries were generated using NEBNext UltraTM RNA Library Prep Kit for Illumina (NEB, USA) following manufacturer's recommendations and index codes were added to attribute sequences to each sample. Briefly, mRNA was purified from total RNA using poly-T oligo-attached magnetic beads. Fragmentation was carried out using divalent cations under elevated temperature in NEBNext First Strand Synthesis Reaction Buffer (5X). First strand cDNA was synthesized using random hexamer primer and M-MuLV Reverse Transcriptase (RNase H-). Second strand cDNA synthesis was subsequently performed using DNA Polymerase I and RNase H. Remaining overhangs were converted into blunt ends via exonuclease/polymerase activities. After adenylation of 3' ends of DNA fragments, NEBNext Adaptor with hairpin loop structure were ligated to prepare for hybridization. In order to select cDNA fragments of preferentially 250~300 bp in length, the library fragments were purified with AMPure XP system (Beckman Coulter, Beverly, USA). Then 3 μl USER Enzyme (NEB, USA) was used with size-selected, adaptor-ligated cDNA at 37°C for 15 m in followed by 5 min at 95 °C before PCR. Then PCR was performed with Phusion High -Fidelity DNA polymerase, Universal PCR primers and Index (X) Primer. At last, PCR products were purified (AMPure XP system) and library quality was assessed on the Agilent Bioanalyzer 2100 system.

**ATAC-seq experiment**

For ATAC-seq, we used the infarct zone tissues of MI and MI+P groups and LV tissue in corresponding site of Sham group in rats 4 weeks post-surgery. We isolated the nuclei from frozen tissues as previously described ^[10]^ at first. Then the TruePrep® DNA Library Prep Kit V2 for Illumina and TruePrep® Index Kit V2 for Illumina were used to construct the sequencing library. We performed the 2×150bp paired-end sequencing (PE150) on an illumina Novaseq™ 6000 (LC-Bio Technology CO., Ltd., Hangzhou, China) following the vendor's recommended protocol. Trim_galore was used to check the quality of the raw sequencing reads. The high-quality reads were then aligned to a reference genome (Rn7) with Bowtie2. Samtools was applied to convert sequence alignment/map (.SAM) format files into binary alignments/maps (.BAM) format, meanwhile purging duplicates. Following this, MACS2 was utilized not only for peak calling and converting the BAM files into a Bedgraph format but also for generating a list of accessible chromatin regions for each sample. Each sample’s various peaks corresponded to genes and positions on chromosome were recognized and annotated with ChIPseeker package in R software. Deeptools was used for creating heatmaps and profiles of read coverage across regions of interest in the genome. To facilitate the visualization process with the Integrative Genomics Viewer (IGV), bedgraph format files were converted to BigWig format files using the wigToBigWig tool.

**RNA Extraction, Reverse Transcription, and quantitative Real-Time PCR**

Total RNA was isolated from rat and pig heart tissues using an TRIZOL method. Specifically, we used the infarct zone tissues of MI, MI+P, MI+shctrl, MI+sh*Piezo1*, MI+P+oectrl and MI+P+oe*Piezo1* groups and LV tissue in corresponding site of Sham group in rats 4 weeks post-surgery. And we used the infarct zone tissues of MI and MI+P groups and LV tissue in corresponding site of Sham group in pigs 8 weeks post-surgery. cDNA was obtained using a EZB Reverse Transcription System (EZBioscience, USA) and analyzed by quantitative Real-Time PCR (qRT-PCR) using SYBR Green (TSINGKE, China). The data were normalized to expression of 18S. The primer sequences are shown in **Supplementary Table 2 and 3.**

**Immunoblotting analysis**

Frozen heart tissues (~50mg) were homogenized in an ice-cold RIPA lysis buffer (Beyotime, China). Heart homogenates were kept on ice for 0.5 h and then were centrifuged at 15000 rpm for 5min at 4 °C, supernatants were collected for immunoblotting. Next ,100ug of total protein was resolved by 6% or 12% sodium dodecyl sulfate-polyacrylamide gel electrophoresis (SDS-PAGE) electrophoresis and transferred onto a polyvinylidene fluoride (PVDF) (IPVH00010, Millipore, Massachusetts, USA) membrane via electroblotting. The PVDF membrane was blocked in 5% bovine serum albumin (BSA) in TBST for 1h at room temperature. Primary antibodies anti-Piezo1 (1:1000, ProteinTech, 15939-1-AP), anti-beta-Tubulin (1:5000, ProteinTech,10094-1-AP) were applied in Primary&Secondary Antibody Diluent for Imunostaining (Yeasen, USA) at 4˚C overnight. Membranes were washed in TBST for 3 times and incubated with HRP-conjugated secondary anti-rabbit secondary antibodies for 1 hour at room temperature.

**Cell lines and culture**

HEK293T cells (human embryonic kidney) and H9C2 cells were obtained from the ATCC and cultured in Dulbecco's modified Eagle's medium (DMEM) with 10% fetal bovine serum and 1% penicillin and streptomycin at 37°C supplemented with 5% CO_2_. HEK293T cells were transfected with indicated plasmids with GeneTwin transfection reagent (Biomed, TG101-01).

**Plasmids, lentivirus production and infection**

In order to knockdown *Piezo1*, short hairpin RNAs targeting *Piezo1* (The sequences are shown in Supplementary Table 3) were cloned into pLKO.1 vector under the control of a U6 promoter. Lentivirus was produced in HEK293T cells. The day before transfection, 2^10^7^ cells were seeded on a 15 cm culture dish with growth medium. Cells were co-transfected with the lentivirus constructs vector plasmid or above plasmid, psPAX (Addgene,12260), pMD2.G (Addgene,12259) and the ratio of these plasmids is 4:3:1. Lentivirus supernatant was collected at 48h and 72h post transfection. H9C2 cells were used to test the efficiency of knockdown and sh*Piezo1*-#1 was chosen for the following study. The mixture of lentivirus supernatant was concentrated via ultracentrifugation into pellet which was resuspended with cold PBS. Lentivirus targeting *Piezo1* (sh*Piezo1*) or scramble shRNA (shCtrl) were injected into the male Sprague Dawley rats aged 8-10 weeks at the dosage of 10^8^ transducing units (TU) in a final volume of 50 ul immediately post-MI.

The CRISPR-dCas9 activation system was described as a published protocol ^[11]^. This system has two compositions including pLenti-U6-gRNA and pLv-dCas9-VP64 (a gift from Dr. Chaochen Wang). To activate endogenous Piezo1, a sgRNA targeting *Piezo1* (The sequences are shown in **Supplementary Table 4**) was cloned into pLenti-U6-gRNA vector. To test the efficiency of activation, we first constructed stably expressed dCas9-VP64 H9C2 cell lines, then used lentivirus containing different sgRNAs to activate the expression of PIEZO1. gRNA1 were used for the following study. For animal study, two lentiviruses were produced respectively as above and were 1:1 mixed in a total 100 uL volume. In our study, the mixture lentivirus-oe*Piezo1*(short as oe*Piezo1*) were injected into the MI site as mentioned above and then a patch was fixed to this area with sutures. And the lentivirus-oeControl (oeCtrl) was used as control.

**Supplementary Figures**

**
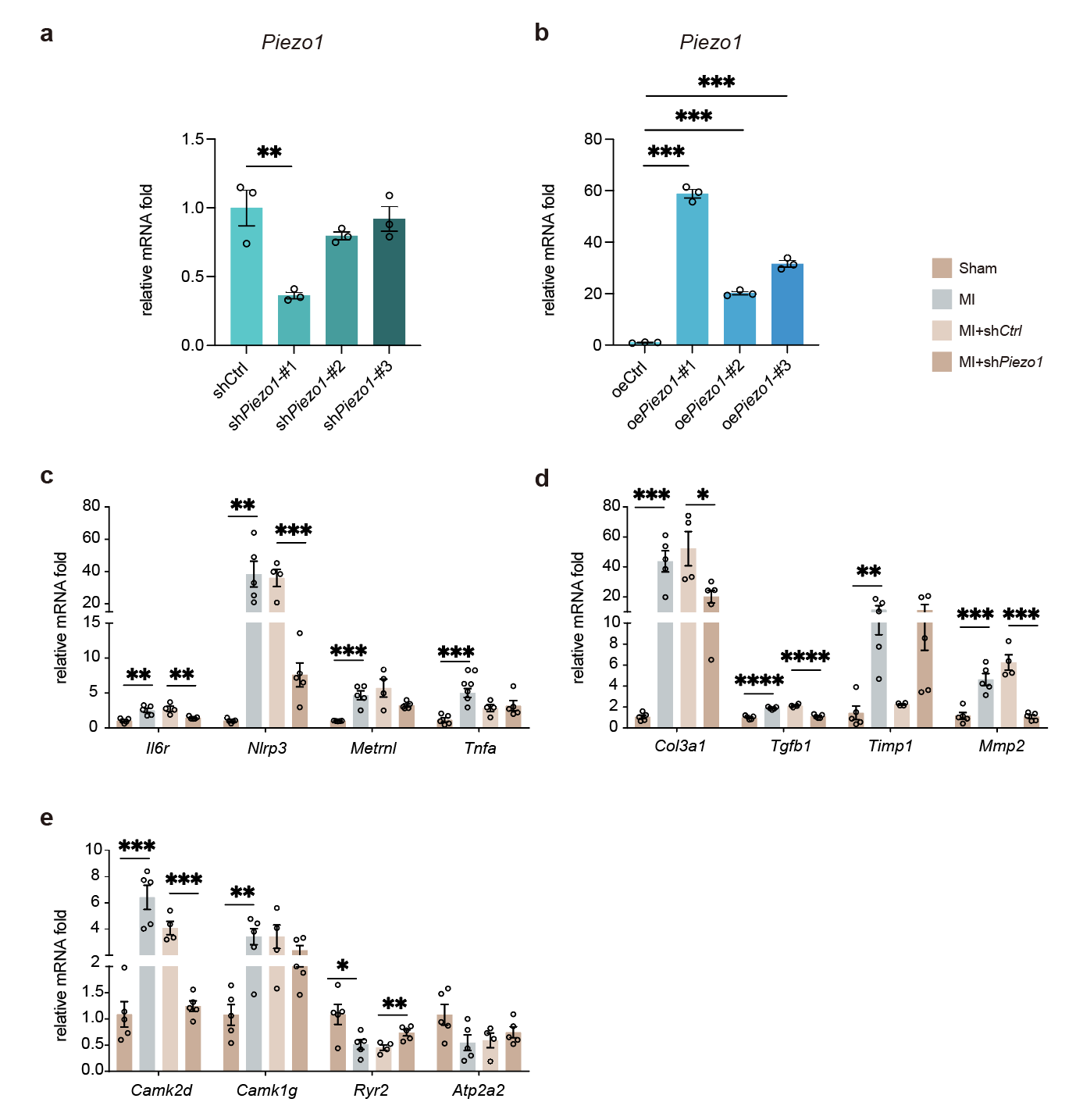
**

**Figure S1 The efficiency of *Piezo1* knockdown and activation by lentivirus.**

(**a**) Quantification of *Piezo1* mRNA expression in H9C2 with scramble RNA or sh*Piezo1.* (**b**) Quantification of *Piezo1* mRNA expression in H9C2 with oeCtrl or oe*Piezo1.* (**c-e**) Expression levels of in (**c**) inflammatory response genes (*IL6r, Nlrp3, Metrnl* and *Tnfa*), (**d**) collagen fibril organization genes (*Col3a1, Tgfb1, Timp1* and *Mmp2*), (**e**) calcium ion transport genes (*Camk2d, Camk1g,Ryr2* and *Atp2a2*) in the groups of Sham, MI, MI+sh*Ctrl* and MI+sh*Piezo1*, determined by qRT-PCR with RNA extracted from the rats.**p* < 0.05, ***p* < 0.01 and ****p* < 0.001 comparison between groups was indicated in figures.

**
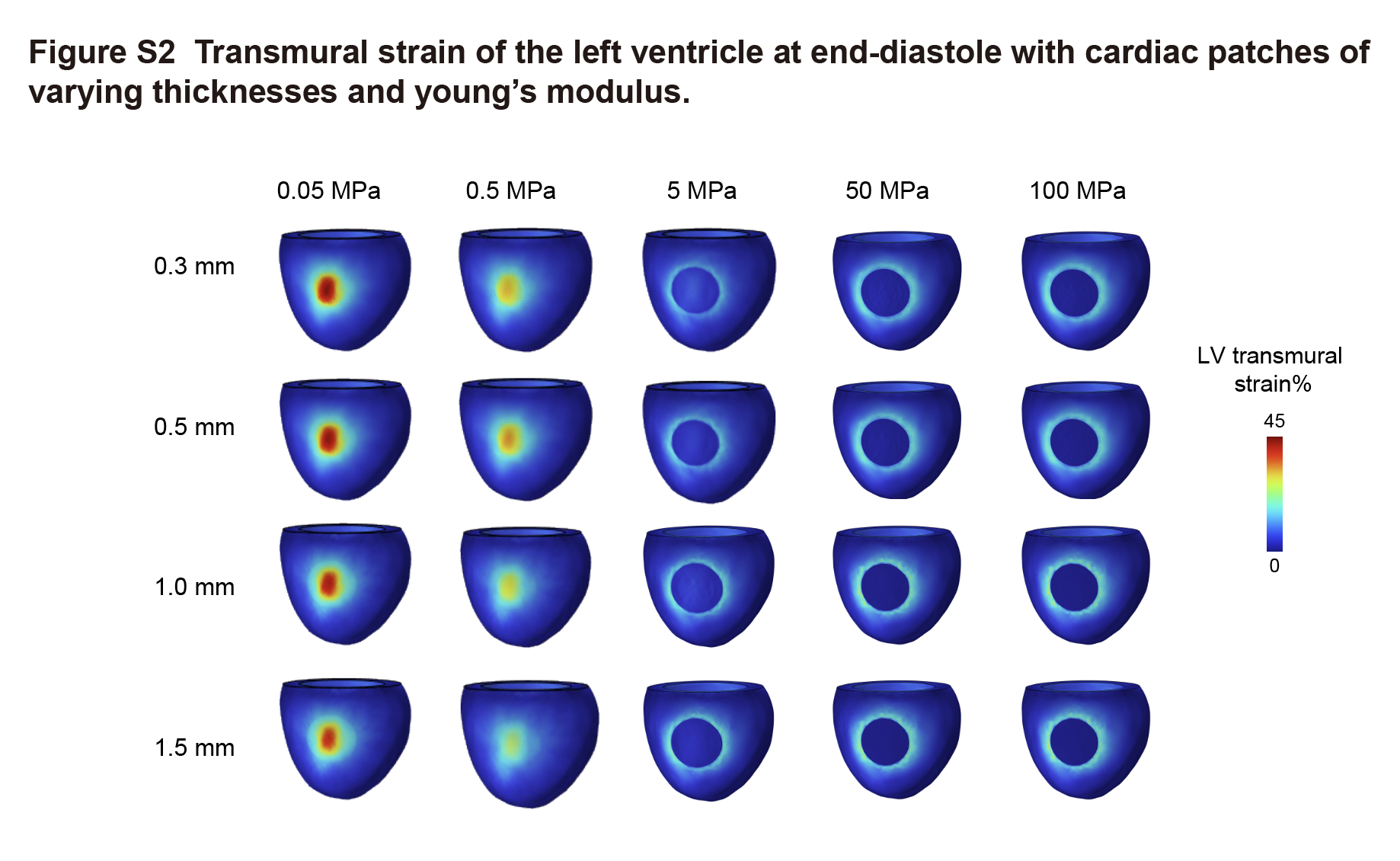
**

**Figure S2 Transmural strain of the left ventricle at end-diastole with cardiac patches of varying thicknesses and young’s modulus.**

**
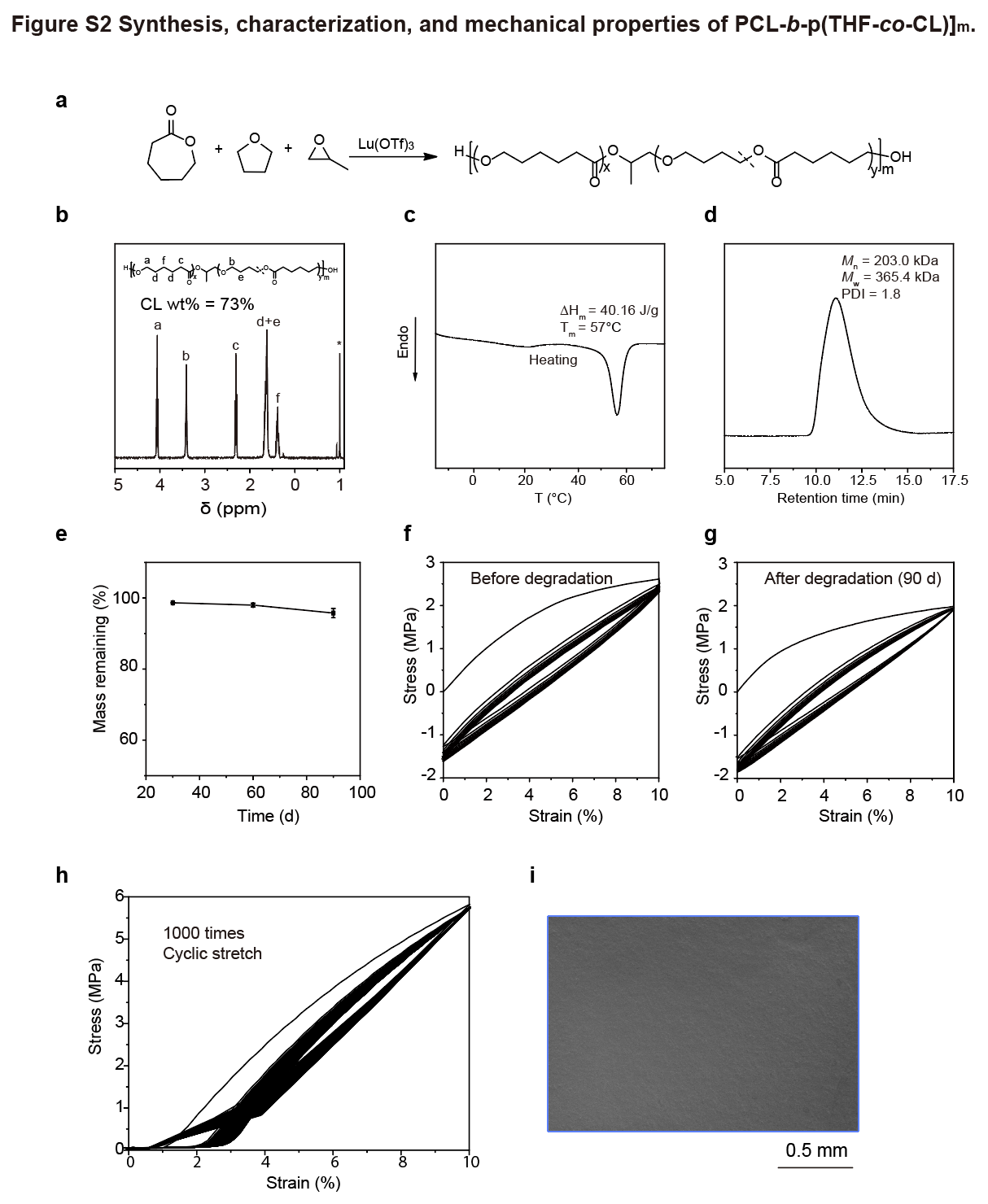
**

**Figure S3 Synthesis, characterization, and mechanical properties of [PCL-*b*-p(THF-*co*-CL)]_m_.**

(**a**)Janus polymerization of CL and THF catalyzed by Lu(OTf)3/PO. (**b**) 1H NMR spectra of [PCL-*b*-p(THF-*co*-CL)]_m_. (**c**) DSC curve of [PCL-*b*-p(THF-*co*-CL)]_m_. (**d**) SEC traces of [PCL-*b*-p(THF-*co*-CL)]_m_ for molecular weight measurement. (**e**) Remaining mass (Wt/W0*100%) of [PCL-*b*-p(THF-*co*-CL)]_m_ after different degradation periods. Cyclic stretch results of cardiac patches at 10% elongation (**f**) before and (**g**) after degradation. (**h**) Stress-strain behavior of [PCL-*b*-p(THF-*co*-CL)]_m_ under 1000 times of cyclic stretch. (**i**) SEM characterization of [PCL-*b*-p(THF-*co*-CL)]_m_ patch surface.

**
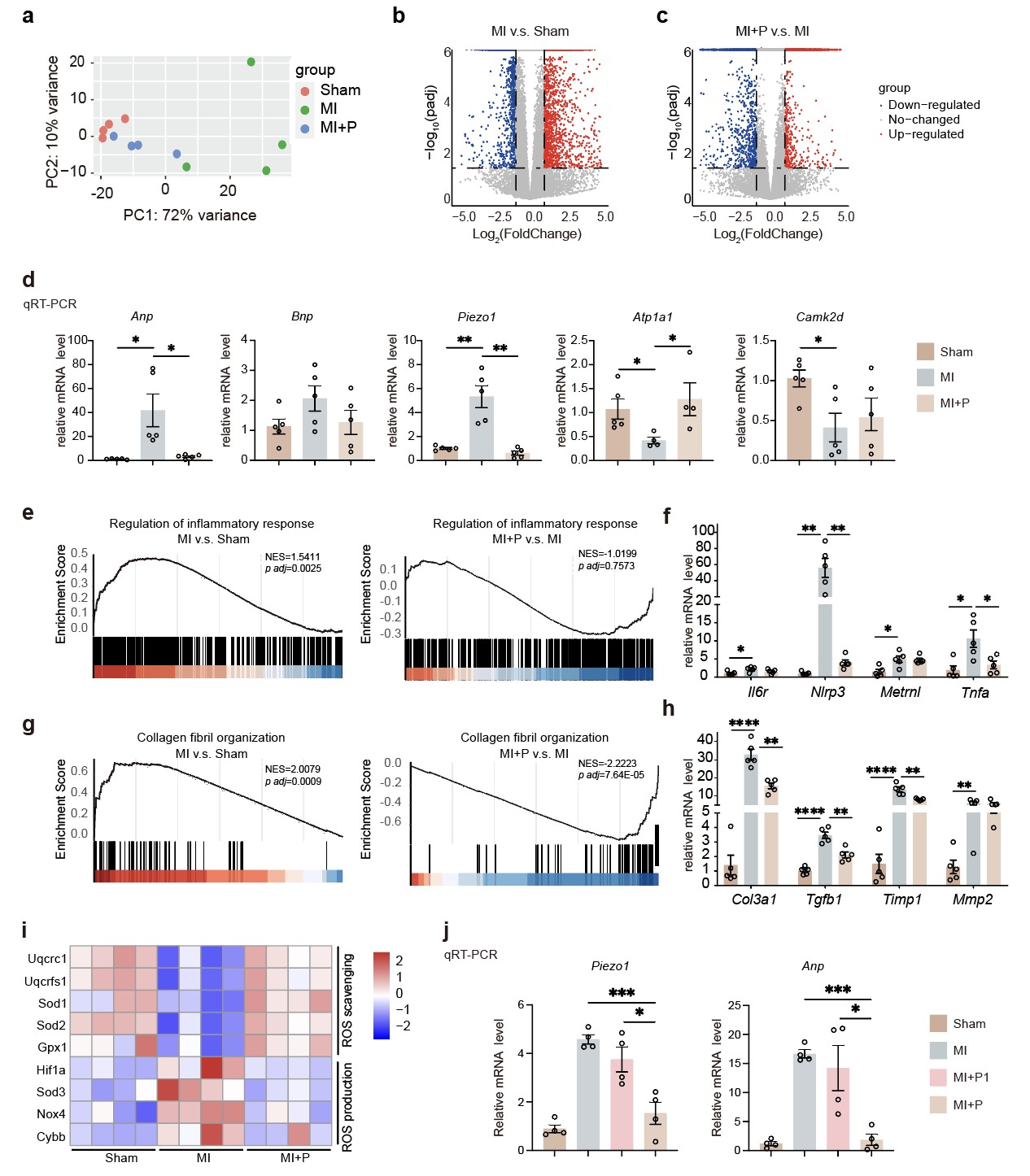
**

**Figure S4 Transcriptomic and qRT-PCR analysis reveal the regulation of *Piezo1* and cardiac related genes in MI rats with patch treatment.**

(**a**) Principal component analysis (PCA) of transcriptomes of the infarct area from the rat groups of Sham, MI and MI+P (n = 4 per group). (**b-c**) Volcano plot of all expressed genes from RNA-seq data comparing MI to Sham (**b**), and comparing MI+P to MI (**c**). Horizontal dash-dot line represents the threshold for adjusted *p* value = 0.05, while vertical dash-dot lines show the value of Log_2_ (FoldChange) being 1. (**d**) Expression levels of *Piezo1, Atp1a1, Anp, Bnp* and *Camk2d* in the groups of Sham, MI and MI+P, determined by qRT-PCR with RNA extracted from the rats. (**e-h**) Gene set enrichment analysis (GSEA) of the genes in (**e**) inflammatory response and (**g**) collagen fibril organization between MI and sham or between MI+P and MI. Expression levels of (**f**) inflammatory response genes (*IL6r, Nlrp3, Metrnl* and *Tnfa*) and (**h**) collagen fibril organization genes (*Col3a1, Tgfb1, Timp1* and *Mmp2*) in the groups of Sham, MI and MI+P. (**i**) The heatmap shows the RNA-seq expression of ROS production and scavenging relative genes. (**j**) Expression levels of *Piezo1* and *Anp* in the groups of Sham, MI, MI+P1, and MI+P, determined by qRT-PCR with RNA extracted from the rats. **p* < 0.05, ***p* < 0.01 and ****p* < 0.001 comparison between groups was indicated in figures.

**
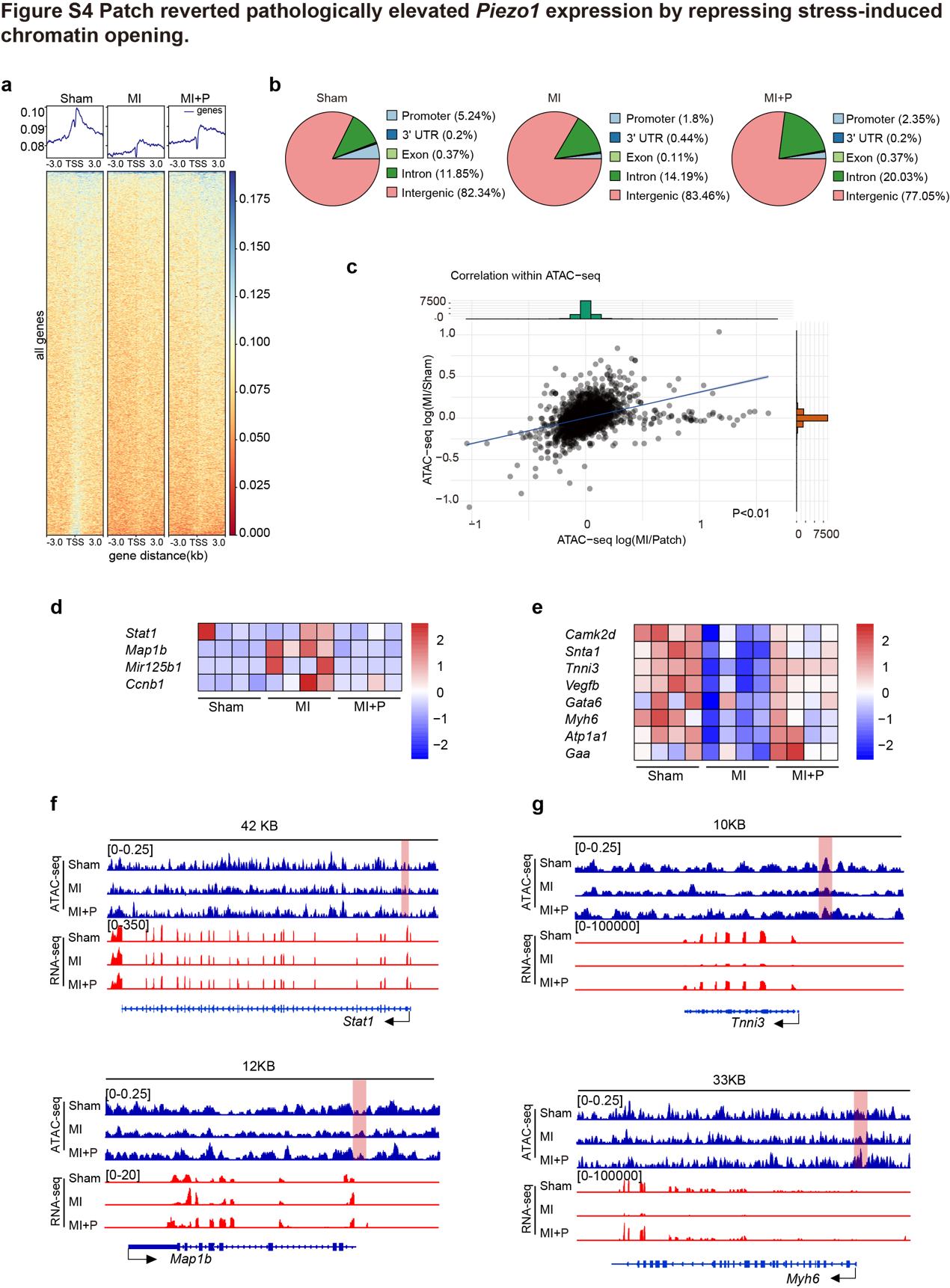
**

**Figure S5 Patch reverted pathologically elevated *Piezo1* expression by repressing stress-induced chromatin opening.**

(**a**) The overall first 1/3 peak abundance in the promoter regions. (**b**) The distribution of ATAC-seq peaks on chromosomes in Sham, MI, MI+P samples. (**c**) Correlation between the ratios of MI/Sham and MI/MI+P in ATAC-seq analysis. Each point in the graph reflected the ratio of the gene's ATAC-seq reads in MI/Sham or MI/MI+P. Ratios of all known genes were presented logarithmically. (**d-e**) The heatmap shows the RNA-seq expression of mechanical stimulus (**d**) and heart contraction (**e**) related genes within patch effective peaks. (**f-g**) Snapshot showing peaks of Stat1, Map1b, Tnni3, Myh6 with or without patch treatment of ATAC-seq and RNA-seq.

**
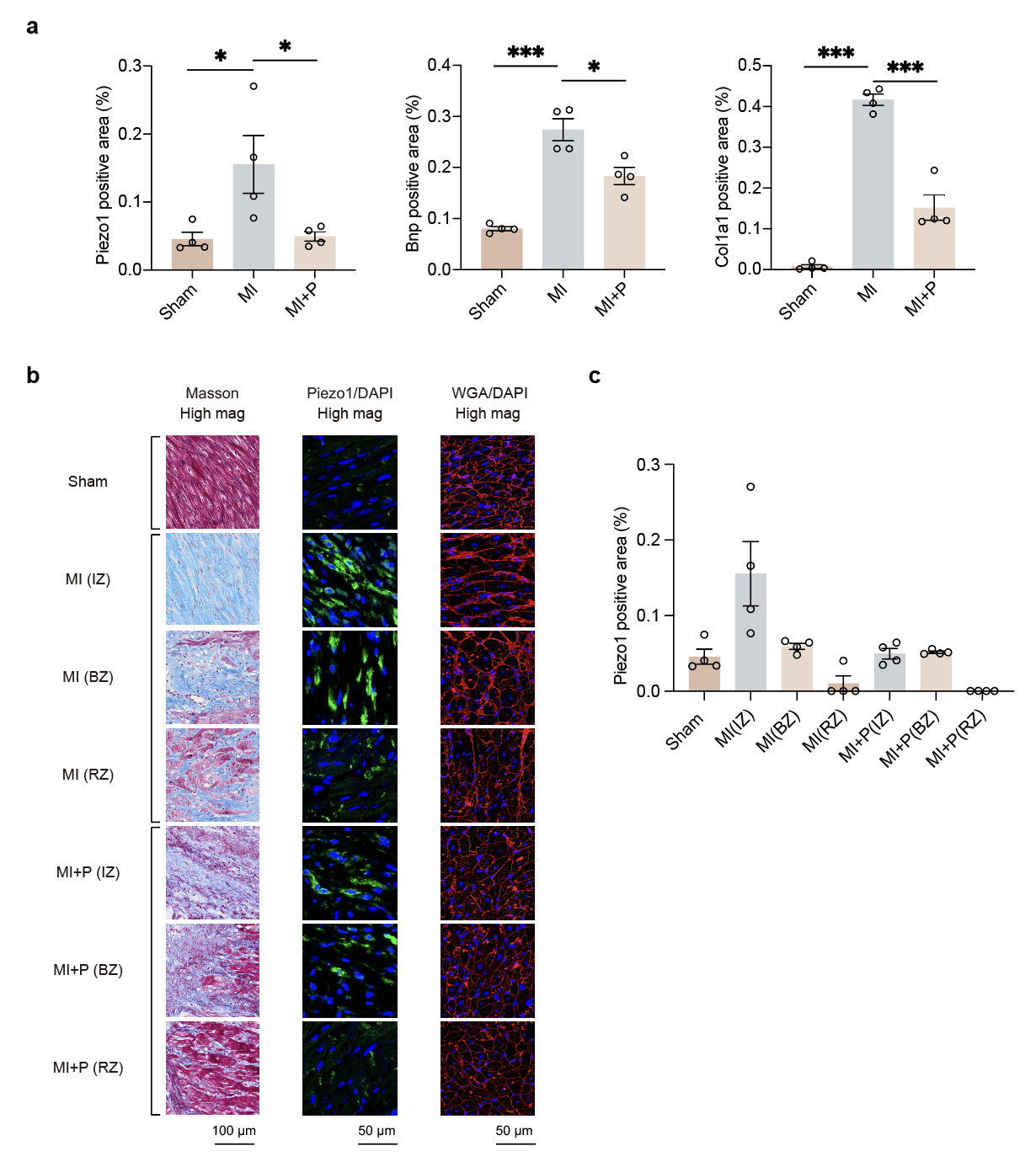
**

**Figure S6 Reverting stress-induced *Piezo1* expression by patch implantation improved LV function and limited LV remodeling in porcine model.**

(**a**) Quantification analysis of Piezo1, Bnp, Col1a1 positive area in IZ. (**b**) Representative histological analysis of IZ, BZ, RZ in three groups: Masson’s trichrome staining and immunofluorescence staining for Piezo1 (green), WGA (red). (**c**) Quantification analysis of Piezo1 positive area in IZ, BZ, and RZ. IZ = infarcted zone; BZ = border zone; RZ = remote zone. All data are means ± SEM. **p* < 0.05, ***p* < 0.01 and ****p* < 0.001 comparison between groups was indicated in figures.

**Supplementary Tables**

**
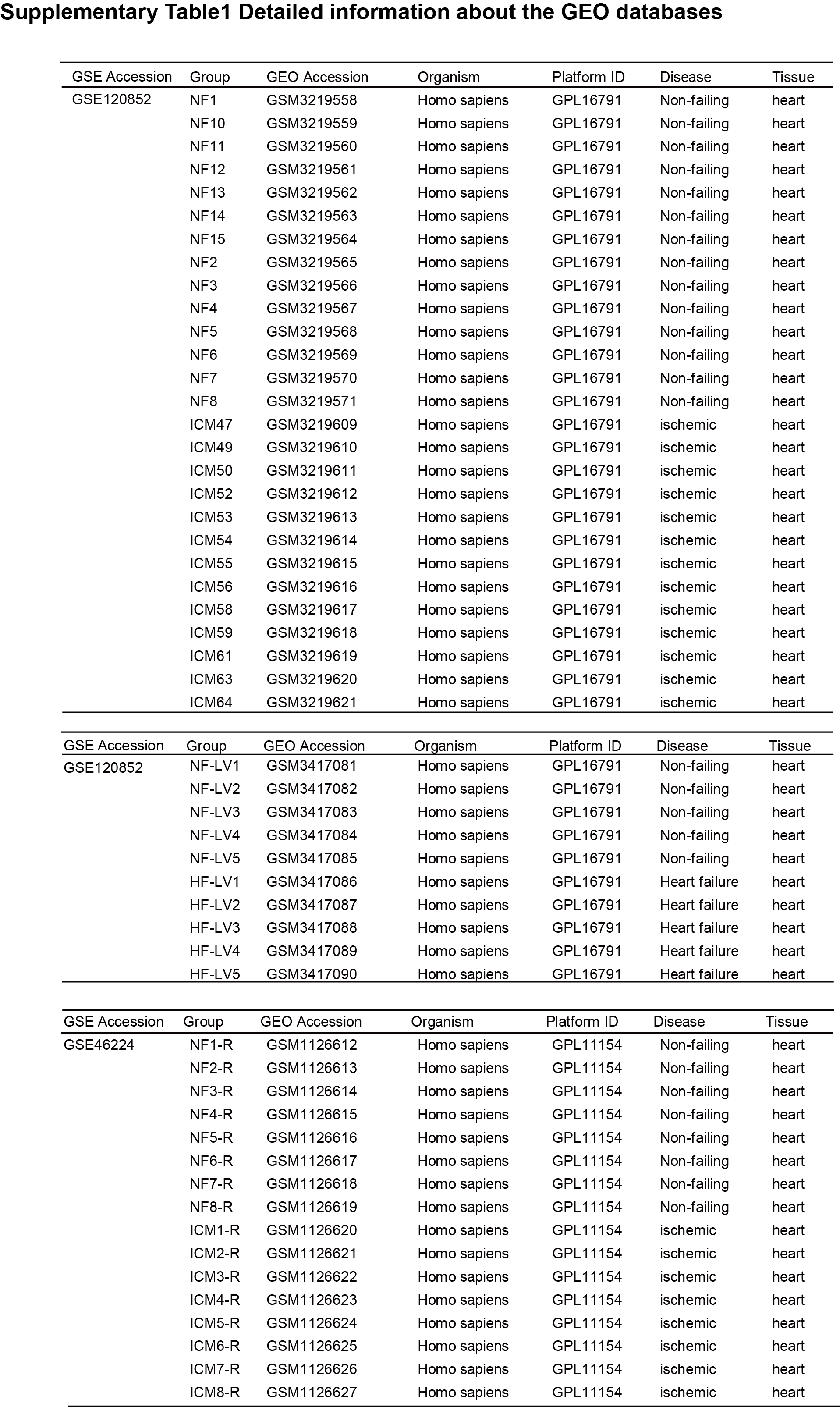
**

**
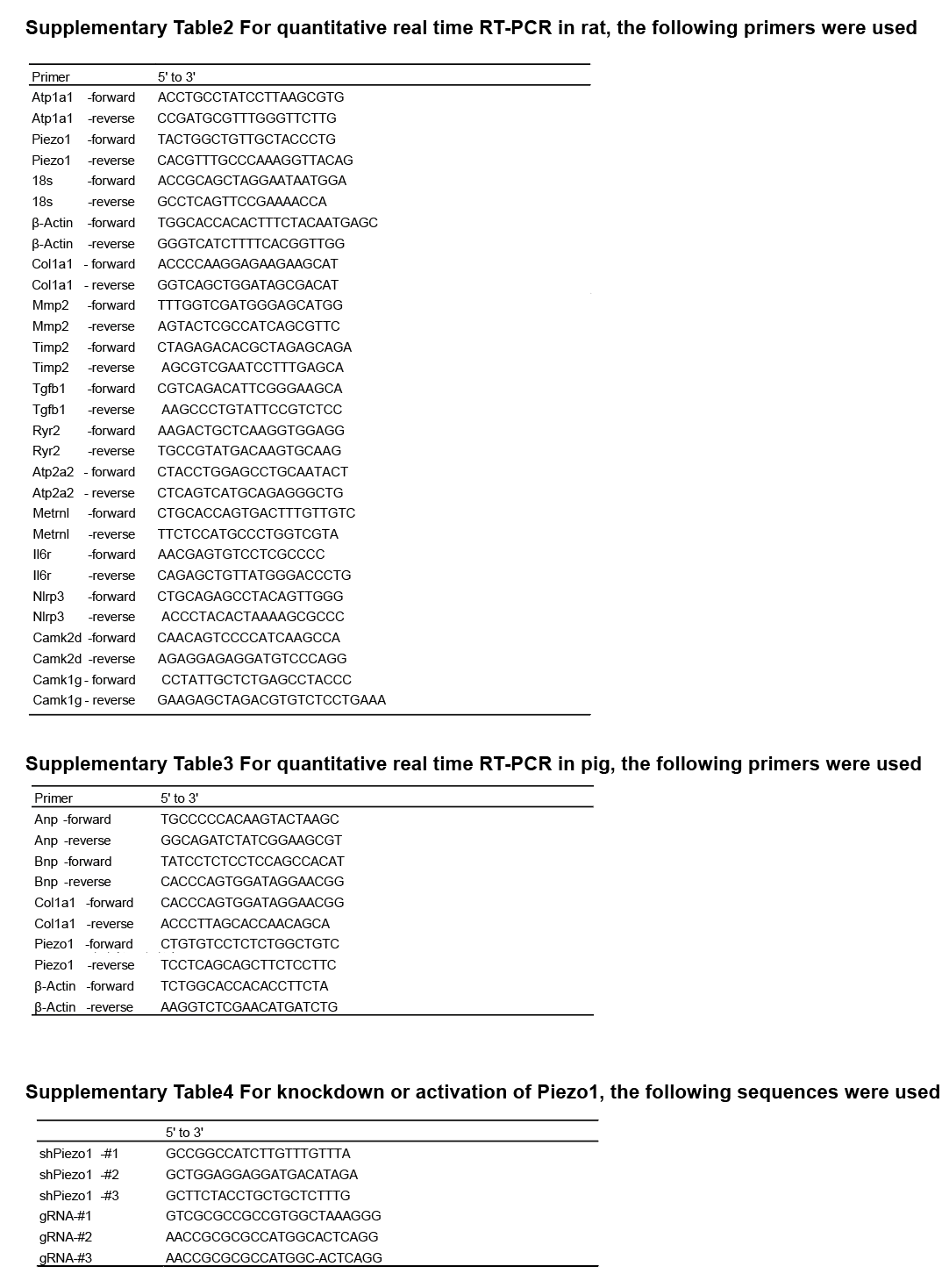
**

**Supplementary Reference**

[1] D. Kim, J. M. Paggi, C. Park, C. Bennett, S. L. Salzberg, *Nat. Biotechnol.* **2019**, *37*, 907.

[2] H. Li, B. Handsaker, A. Wysoker, T. Fennell, J. Ruan, N. Homer, G. Marth, G. Abecasis, R. Durbin, *Bioinformatics* **2009**, *25*, 2078.

[3] M. I. Love, W. Huber, S. Anders, *Genome Biol.* **2014**, *15*, 550.

[4] M. E. Ritchie, B. Phipson, D. Wu, Y. Hu, C. W. Law, W. Shi, G. K. Smyth, *Nucleic Acids Res.* **2015**, *43*, e47.

[5] S. Wang, C. Qin, N. Savioli, C. Chen, D. P. O’Regan, S. Cook, Y. Guo, D. Rueckert, W. Bai, in *Medical Image Computing and Computer Assisted Intervention–MICCAI 2021: 24th International Conference, Strasbourg, France, September 27–October 1, 2021, Proceedings, Part III 24* Springer, **2021**, 14-24.

[6] H. Gao, W. Li, L. Cai, C. Berry, X. Luo, *Journal of engineering mathematics* **2015**, *95*, 231.

[7] L. You, T. E. Hogen-Esch, Y. Zhu, J. Ling, Z. Shen, *Polymer* **2012**, *53*, 4112.

[8] L. You, J. Ling, *Macromolecules* **2014**, *47*, 2219.

[9] Y. Lu, T. Ren, H. Zhang, Q. Jin, L. Shen, M. Shan, X. Zhao, Q. Chen, H. Dai, L. Yao, J. Xie, D. Ye, T. Lin, X. Hong, K. Deng, T. Shen, J. Pan, M. Jia, J. Ling, P. Li, Y. Zhang, H. Wang, L. Zhuang, C. Gao, J. Mao, Y. Zhu, *Acta Biomater.* **2022**, *153*, 386.

[10] M. R. Corces, A. E. Trevino, E. G. Hamilton, P. G. Greenside, N. A. Sinnott-Armstrong, S. Vesuna, A. T. Satpathy, A. J. Rubin, K. S. Montine, B. Wu, A. Kathiria, S. W. Cho, M. R. Mumbach, A. C. Carter, M. Kasowski, L. A. Orloff, V. I. Risca, A. Kundaje, P. A. Khavari, T. J. Montine, W. J. Greenleaf, H. Y. Chang, *Nat. Methods* **2017**, *14*, 959.

[11] J. Joung, S. Konermann, J. S. Gootenberg, O. O. Abudayyeh, R. J. Platt, M. D. Brigham, N. E. Sanjana, F. Zhang, *Nat. Protoc.* **2017**, *12*, 828.
